# A Folded, Structure‐Integrated Bimodal Sensor Enabling Non‐Contact and Tactile Perception for Intelligent Robots

**DOI:** 10.1002/advs.75868

**Published:** 2026-05-28

**Authors:** Weixiong Yang, Yuhan Guo, Mingguang Han, Bin Feng, Yuan Ma, Bingang Xu, Fu Liu, Dan Wang, Pingping Hao, Xilun Ding, Sida Luo

**Affiliations:** ^1^ School of Mechanical Engineering & Automation Beihang University Beijing China; ^2^ Department of Mechanical Engineering The Hong Kong Polytechnic University Hong Kong SAR China; ^3^ Nanotechnology Center School of Fashion and Textiles The Hong Kong Polytechnic University Kowloon Hong Kong China; ^4^ School of Aerospace Engineering North University of China Taiyuan China

**Keywords:** bimodal sensor, integrated structure, intelligent robotic system, laser‐induced graphene, triboelectric nanogenerators

## Abstract

The rapid advancement of embodied intelligent robots demands accurate perception and integration of diverse environmental cues. However, current multimodal sensing systems are hindered by complex architectures and heterogeneous integration, which compromise rapid fabrication, seamless deployment, robustness, and multi‐source signal perception. Here, we address this challenge through an elegant substrate‐folding design strategy that enables bimodal sensing within a single monolithic laser‐induced graphene (LIG) film, drastically simplifying device architecture and fabrication while paving the way for scalable, robot‐ready multimodal perception modules. The folded bimodal sensor (F‐BS), which features a one‐step fabrication and assembly process, enables real‐time detection of both non‐contact proximity signals and contact tactile signals with mode differentiation by leveraging the triboelectric nanogenerator (TENG) effect and the piezoresistive property of graphene. After fluorination, it exhibits a non‐contact sensing distance of 110 mm, a maximum pressure sensitivity of 11.2 kPa^−1^, and a response time of 10 ms. The integration of proximity‐tactile information enables an intelligent robotic system capable of accurately characterizing objects based on their physical properties, such as orientation, material, and hardness, with a recognition accuracy of up to 99% regardless of ambient lighting. This work provides a hardware foundation and expanded possibilities for human‐robot interface systems with intelligent interaction capabilities.

## Graphical abstract

A foldable bimodal sensor integrates a triboelectric nanogenerator and graphene piezoresistive effect into a monolithic laser‐induced graphene film. It enables synchronous non‐contact and tactile perception, achieving a distance of 110 mm, a sensitivity of 11.2 kPa^−^^1^, and a response time of 10 ms. Integrated into an intelligent robotic system, it recognizes object properties with 99% accuracy for reliable high‐risk grasping in dark environments.

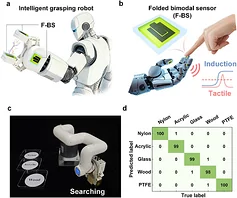

## 1 Introduction

With the advent of artificial intelligence, cutting‐edge robots equipped with human‐like sensory capabilities have garnered increasing attention [1, 2, 3, 4, 5]. Research on environmental perception has driven advancements in robotic technology, endowing robots with exceptional sensing capabilities for object detection, threat recognition, and precise manipulation [6, 7, 8]. Currently, the aforementioned sensing modalities are typically realized by tactile sensors capable of responding to diverse stimuli. For instance, existing terminal sensor technologies—including capacitive [9, 10], piezoresistive [11, 12], triboelectric [13], pyroelectric [14], giant magnetoelastic [15], and piezoelectric [16] types—can detect physical information such as pressure, distance, and temperature. Although these sensors enable robots to perceive specific types of physical information, their unimodal operating characteristics fail to meet the requirements for multi‐level intelligent behaviors in cutting‐edge robots, especially amid increasingly demanding and complex working environments [17]. A critical gap remains in the field of intelligent manipulators regarding multi‐source information perception and parallel processing technologies, which restricts their ability to perceive multimodal environmental information and execute precise operational tasks [18, 19]. To expand the practical application scope of existing robots, the development of high‐performance multimodal sensors and intelligent systems integrated with machine learning has become increasingly urgent.

In response to this need, a growing body of research has been dedicated to the development of multimodal end‐effector sensors for object perception and feature recognition. Some scholars have proposed sensors based on a single functional material substrate or fabrication method [20, 21, 22]. For instance, modified triboelectric components [23, 24], liquid metal encapsulation with specially designed configurations [25], and capacitive sensors [26, 27] have been employed to achieve proximity and contact detection. However, significant technical challenges persist in decoupling and distinguishing different stimulus signals. Other researchers have focused on expanding the diversity of functional materials and achieving multimodal sensing by adopting a single conversion mechanism for each material [28, 29, 30]. For instance, flexible multimodal sensors based on triboelectric nanogenerators and liquid metals [31], as well as multiplexed electronic skins, have demonstrated multimodal sensing of stretching, compression, and touch [32]. Although these sensors have achieved preliminary decoupling of multimodal information, they often suffer from excessively large and complex structures with redundant layers, while also relying on sophisticated processing techniques and stringent fabrication conditions [33, 34]. These issues lead to unstable interfaces, poor responsiveness, and unsuitability for mass production. Therefore, the development of a monolithic dual‑mode sensor featuring rapid fabrication, a simple structure, high sensitivity, and the capability to extract diverse object information with easy decoupling has become an important complement to existing robotic perception technologies.

In this study, we developed a folded bimodal sensor (F‐BS) based on the triboelectric nanogenerators and the graphene piezoresistive effect, which achieves two core functions: non‐contact sensing and tactile perception. This capability significantly enhances the interaction ability and operational performance of robots with the physical world. Through rapid integrated processing based on laser‐induced graphene technology (LIG) [35, 36, 37, 38], fluorinated polyimide (F‐PI) film substrate was precisely patterned to form specially arranged graphene electrodes, and combined with a folded structural design, a monolithic device featuring high integration, rapid manufacturability, simple structure, and high sensitivity was ultimately fabricated [39, 40]. Thus, the sensor can simultaneously detect signals in both modes, with a non‐contact sensing distance of 110 mm, a maximum tactile perception sensitivity of 11.2 kPa^−1^, and a minimum response time of 10 ms. Through the integration and training of diverse object sensing data via machine learning algorithms, this intelligent robotic system can accurately recognize object features with a recognition accuracy of up to 99%, enabling object classification and vision‑free hazardous operations. The recognition accuracy is at the same leading level as that of advanced sensor devices in the field [3, 29, 41, 42, 43, 44, 45, 46]. In summary, this work presents a bimodal sensor with an integrated processing and folding structure, which significantly expands the interaction capability between robots and the environment across various application scenarios. It is anticipated to unlock extensive application prospects in detection robots, intelligent prosthetics, and human‐machine interface.

## 2 Results and Discussion

### 2.1 Structural Design and Working Mechanism

**FIGURE 1:**
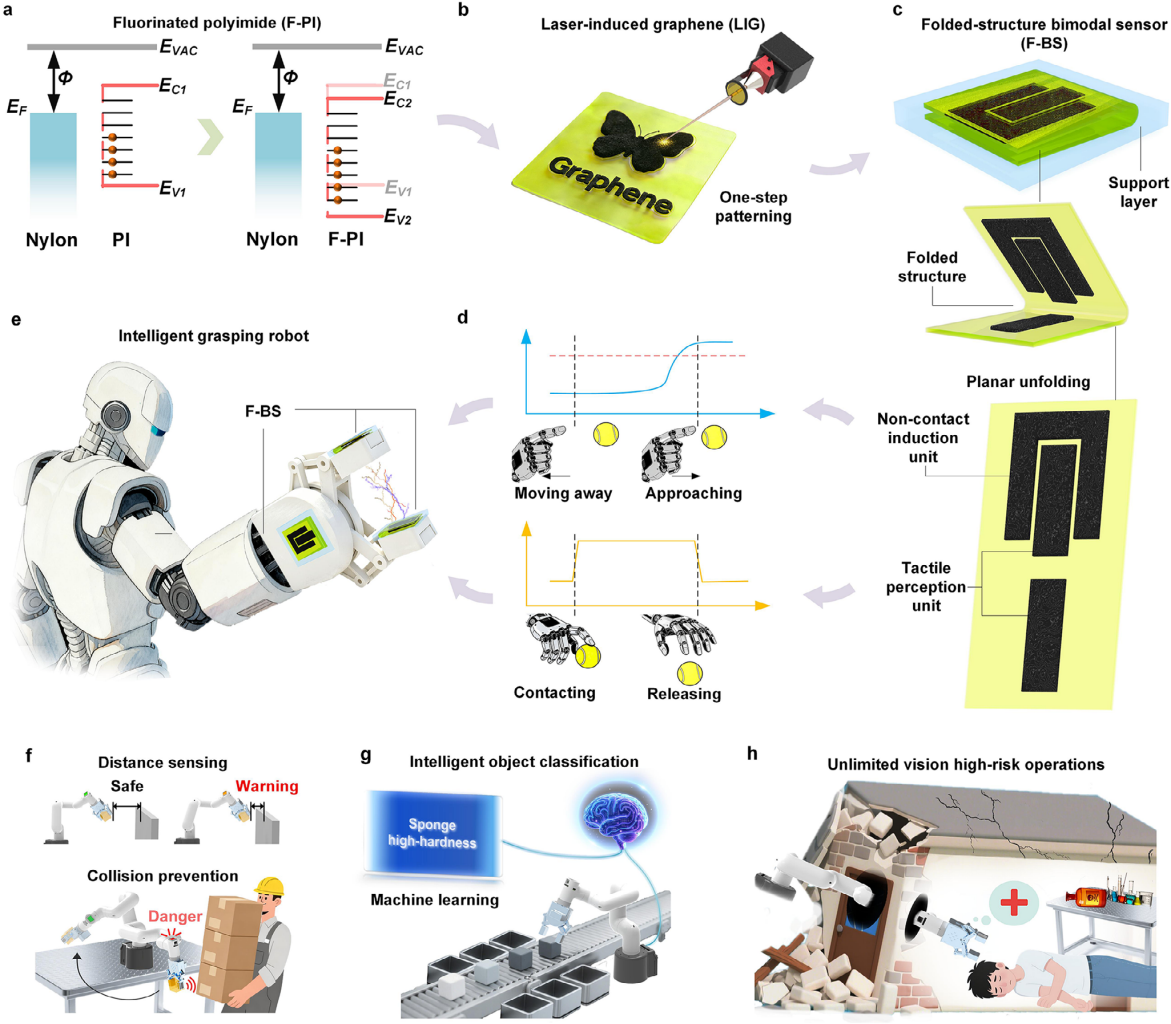
Schematic diagrams of the F‐BS and the integrated intelligent grasping robot. (a) Modified surface states model of PI and fluorinated PI. (b) One‐step laser‐induced graphene (LIG). (c) Schematic diagram of the structure of the F‐BS. (d) Signal demonstration of the non‐contact induction unit and the tactile perception unit. (e) Schematic diagram of the non‐destructive installation of the bimodal sensor on the grasping robot. (f) Distance recognition of obstacles and hazard avoidance by the intelligent grasping robot. (g) Object material and hardness classification system based on machine learning. (h) High‐risk rescue operations without visual field in extreme environments.

First, a fluorinated polyimide (F‐PI) film with high electronegativity was adopted as the substrate (Figure 1a). This substrate was subsequently processed via laser‐induced graphene (LIG) technology, a rapid, one‐step, and programmably customizable approach, to form a monolithic device with an extremely simple structure (Figure 1b). Based on the two advanced technologies described above, the overall design of the proposed folded bimodal sensor (F‐BS) is shown in Figure 1c. It features a nested compact configuration that integrates the non‑contact induction unit and the tactile perception unit, while Ecoflex serves as the support layer. The folded structure of the F‐BS is progressively unfolded to reveal that the non‑contact induction unit is formed by the peripheral graphene electrodes and the F‐PI layer, while the tactile perception unit is composed of the upper and lower strip‐shaped graphene electrodes. The non‑contact induction unit is responsible for detecting object features, including distance and material type, whereas the tactile perception unit measures contact pressure, hardness, and related properties (Figure 1d). By seamlessly integrating F‐BSs into a robotic gripper, this work realizes an anthropomorphic robotic gripper with exceptional multimodal perception capabilities (Figure 1e). The F‐BS is capable of identifying object orientation and avoiding hazardous obstacles (Figure 1f), while also recognizing material type, contact status, and hardness for object sorting and classification (Figure 1g). Additionally, the system enables obstacle avoidance in dark environments, precise localization of hazardous materials, and assists in object retrieval and rescue operations (Figure 1h).

**FIGURE 2:**
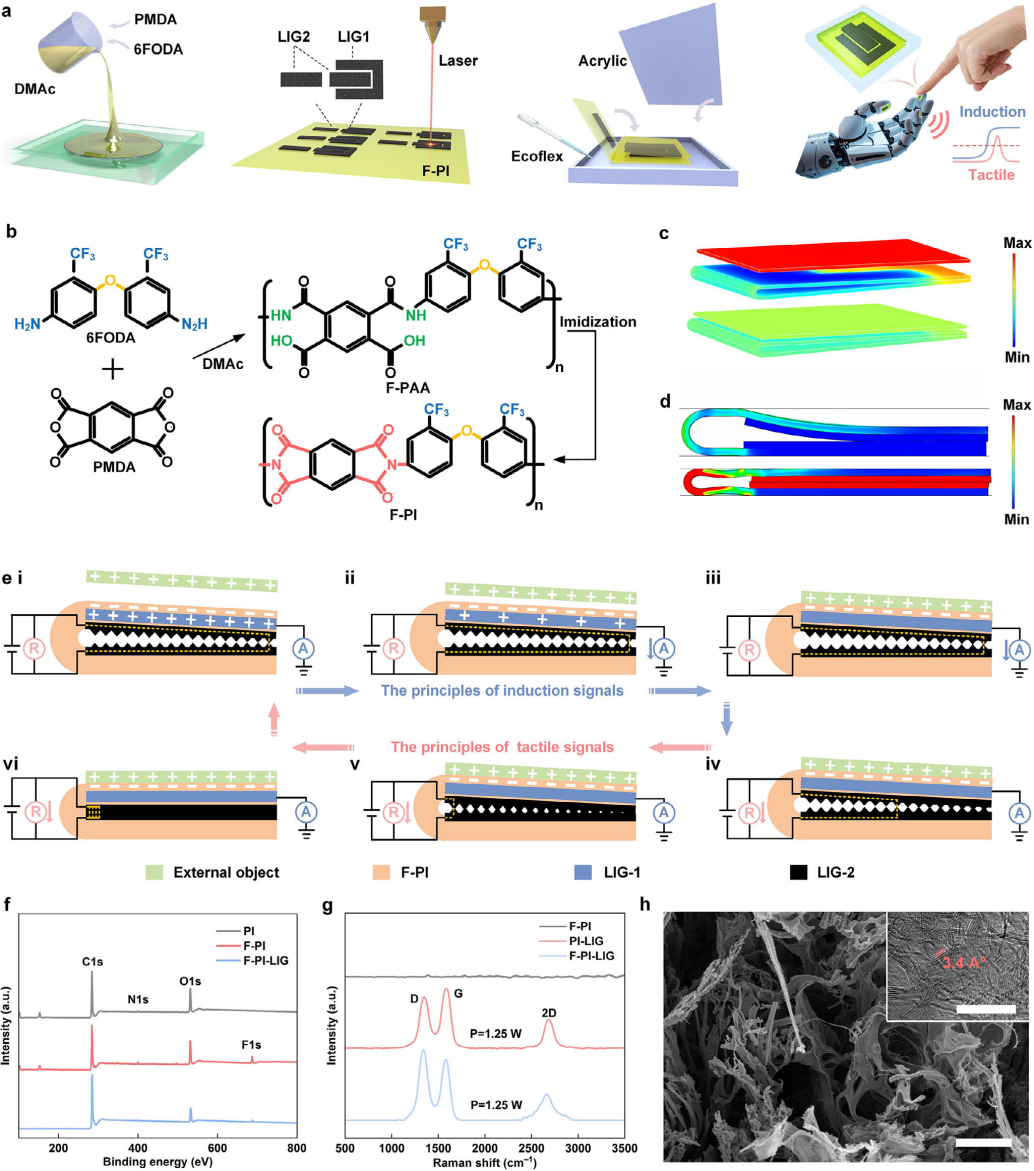
Structural design, fabrication process, and working principles of the F‐BS. (a) Structural design and fabrication flowchart of the F‐BS. (b) Schematic illustration of the synthesis mechanism of F‐PI. (c) Electric field distribution of the triboelectric generation principle at different positions of the external object. (d) Compression process and mechanical simulation of the folded structure. (e) Working principle diagrams for the two sensing modes of the F‐BS. (f) XPS analysis of pristine PI, F‐PI, and F‐PI‐LIG. (g) Raman analysis of F‐PI, PI‐LIG, and F‐PI‐LIG. (h) SEM (scale = 10 µm) and TEM (inset, scale = 10 nm) images of F‐PI‐LIG.

First, pyromellitic dianhydride (PMDA) was mixed with 4,4'‐diamino‐2,2'‐bis (trifluoromethyl) biphenyl (6FODA) and 4,4'‐oxydianiline (ODA) in N,N‐dimethylacetamide (DMAc), a polar aprotic reaction solvent (Figure 2a). An F‐PI film (thickness: 100 µm) was prepared via room‐temperature stirring and thermal imidization. Multiple graphene electrodes were simultaneously printed and cut using LIG technology to obtain monomer components. The folded monomer components were placed into an acrylic mold, and the final bimodal sensor (dimensions: 20 mm × 20 mm × 0.7 mm) was formed by Ecoflex casting and curing. This sensor can be non‐destructively mounted on a humanoid robotic gripper, enabling both non‐contact induction and tactile perception functions. In contrast to recent studies, this sensor is fabricated from a single film in a one‐step process (Table S4). The synthesis mechanism of F‐PI, involving the polymerization of monomers into the F‐PAA precursor followed by imidization, is shown in Figure 2b. Fluorine possesses strong electronegativity, which enhances the electron affinity of the material, macroscopically manifesting as the superior electrical performance of F‐PI [47, 48, 49, 50]. The corresponding modified surface states model is presented in Figure S1. In this work, the triboelectric effect of the non‐contact induction unit is further validated via multiphysics analysis (COMSOL). Different positions of the external object correspond to distinct electric potential fields (Figure 2c; Figure S2a). Meanwhile, the closed state and mechanical properties of the folded structure under external force are simulated (Figure 2d; Figure S2b). Specifically, the non‐contact induction function is realized based on the triboelectric generation principle, while the tactile perception function is achieved through the piezoresistive effect of the folded structure and graphene electrodes. Via electrostatic induction, LIG‐1 generates an equal number of positive charges to stabilize the electric potential (Figure 2ei). As an external object approaches, the F‐PI film and the object gradually establish a stable potential, causing positive charges in LIG‐1 to deplete and inducing a current in the external circuit (Figure 2e,ii,iii). Upon contact with the F‐BS, the non‐contact induction process terminates, and tactile perception commences. By virtue of the ingenious design of the folded structure, LIG‐2 exhibits a slight inclination, which constrains the current in the graphene electrode to traverse solely the longest path, thus maximizing the resistance of the tactile perception unit. As the external object further compresses the F‐BS, the inclination angle of the folded structure diminishes with increasing external force until full closure (Figure 2e,iv,v), shortening the conduction path of current and reducing the resistance of the perception unit. Notably, when an extreme external force is applied to the sensor surface, the LIG‐2 undergoes further compression (Figure 2e,vi). Owing to the inherently loose and porous structure of the graphene electrode, the compressed LIG‐2 forms numerous current microchannels, leading to a further reduction in sensor resistance.

Furthermore, material structures were characterized via Raman spectroscopy, X‐ray photoelectron spectroscopy (XPS), scanning electron microscopy (SEM), and transmission electron microscopy (TEM), confirming that F‐PI enhances triboelectric generation performance while facilitating the formation of the porous structure of graphene. First, post‐irradiation XPS spectra (Figure 2f) reveal that F‐PI exhibits an increase in fluorine content compared to pristine PI (from 0.42% to 6.67%). After laser processing, the carbon content in the laser‐induced graphene electrodes based on fluorinated polyimide (F‐PI‐LIG) increases significantly (from 72.74% to 90.42%), while the oxygen (18.05%) and fluorine (6.67%) contents decrease to 7.49% and 1.47%, respectively. Additional elemental information is available in the Supporting Information (Figure S3). Figure 2g shows that, in contrast to pristine PI with no characteristic Raman shifts, laser‐induced graphene electrodes based on polyimide (PI‐LIG) prominently exhibit a D peak (≈1345 cm^−1^), G peak (≈1585 cm^−1^), and 2D peak (≈2687 cm^−1^), which reflect the intrinsic properties of few‐layer graphene [51]. In F‐PI‐LIG, the D peak intensity increases while the 2D peak intensity decreases slightly, indicating more impurities in graphene due to the introduction of fluorine. XRD patterns in the Supporting Information further confirm that the graphene electrode has a few‐layer porous structure, with strong peaks (002) and (100) centered at 2θ = 24.3 and 2θ = 43.3, respectively (Figure S4). Notably, high‐magnification SEM images clearly reveal the surface morphology of LIG, showing a foam‐like structure with uniformly distributed porous features. TEM images further unveil the corrugated structural characteristics, confirming the ultrathin nature of LIG with a lattice spacing of ≈3.4 Å (Figure 2h). Additional SEM and TEM images at different magnifications are provided in the Supporting Information (Figure S5‐6).

### 2.2 Performance and Optimization of the F‐BS

**FIGURE 3:**
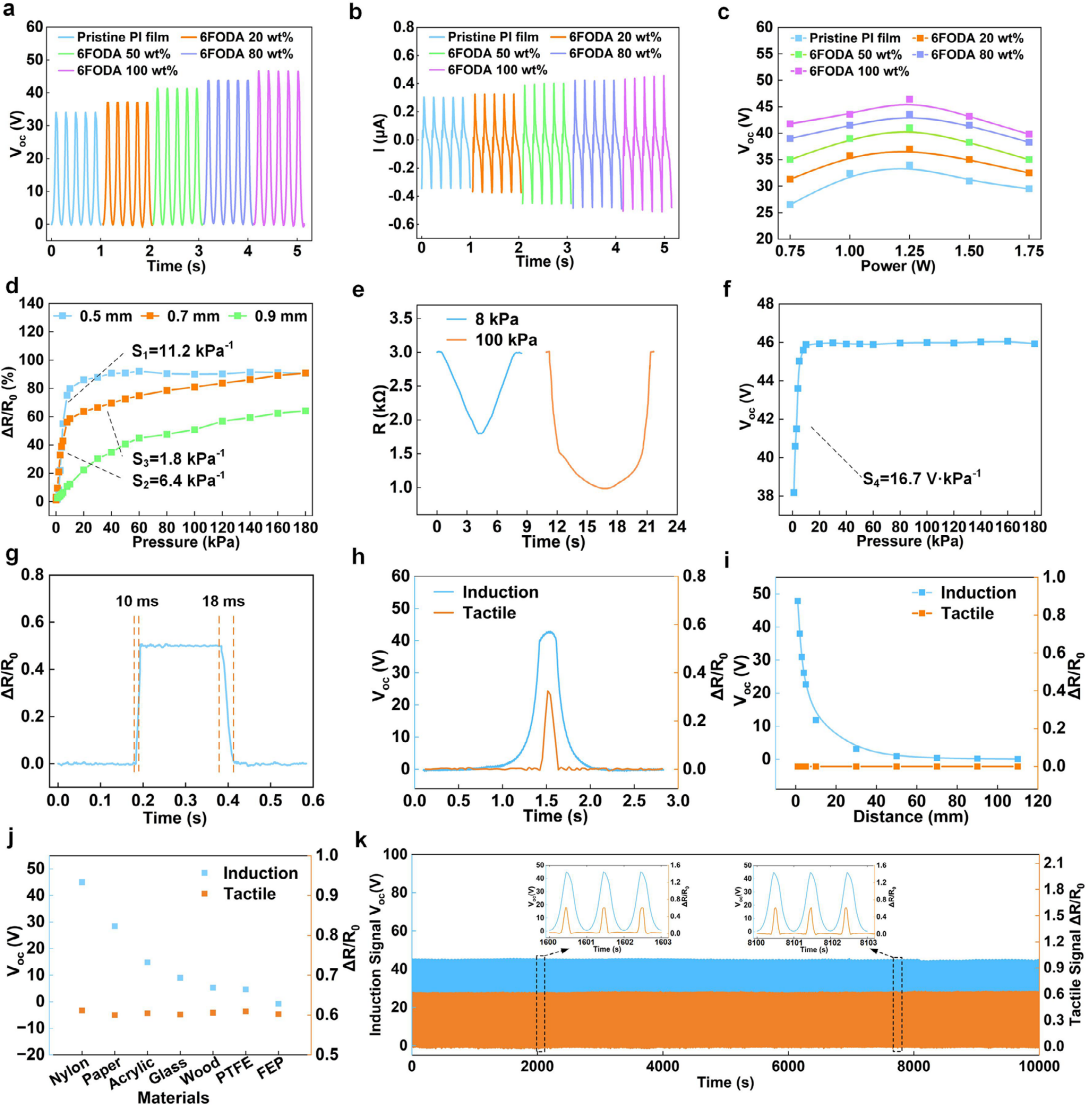
Performance optimization and signal characteristics of the non‐contact induction unit and tactile perception unit of the F‐BS. (a) Voltage performance comparison of TENGs based on pristine PI and F‐PI with different 6FODA contents. (b) Current performance comparison of TENGs based on pristine PI and F‐PI with different 6FODA contents. (c) Line chart of F‐PI performance comparison under different laser powers and different 6FODA contents. (d) Tactile perception performance under different pressures with different mold thicknesses. (e) Tactile perception signal characteristics under low‐pressure and high‐pressure conditions. (f) Voltage performance comparison of TENGs under different pressures. (g) Signal sensitivity of the tactile perception unit. (h) Signal characteristics of the two units of the F‐BS. (i) Performance comparison of the two sensing units at different distances. (j) Signals of F‐BS when in contact with different materials. (k) 10000‐cycle durability test.

The non‐contact induction unit of the F‐BS is essentially based on the fluorinated polyimide (F‐PI)‐based triboelectric nanogenerator (TENG), while its tactile perception unit relies on the piezoresistive property of the folded structure and the graphene structure. Among these, the performance of TENG is determined by the electronegativity of triboelectric materials, and fluorine doping serves as an excellent method to enhance the electronegativity of materials, thereby improving TENG performance. During the fabrication of F‐PI, 6FODA was mixed into ODA at different ratios (20, 40, 60, 80, 100 wt.%) to verify the effect of fluorine elements on TENG performance. The voltage performance and current performance of TENG increase with the rising proportion of 6FODA, with the maximum open‐circuit voltage (Voc) and maximum current (I) reaching 46 V and 0.42 µA, respectively (Figure 3a,b). A voltage performance enhancement of approximately 35.3% is achieved compared to pristine PI. The effect of laser power for graphene electrode processing on TENG performance was further investigated, and the TENG voltage performance under different laser powers and different 6FODA proportions was obtained and plotted into curves (Figure 3c). As the laser power increases, the performance of TENG first increases and then decreases, which is attributed to the differences in the quality of graphene electrodes and the thickness ratio between the electrodes and the F‐PI film [51]. The processing conditions of 1.25 W laser power and 100 wt.% 6FODA content maximize the performance of TENG, which are the optimal processing parameters for the non‐contact induction unit.

Furthermore, molds with different thicknesses (0.5, 0.7, 0.9 mm) were used to adjust the thickness of the gap in the folded structure within the tactile perception unit, and Ecoflex was employed for encapsulating F‐BS and serving as an elastic substrate (Figures 2a and 3d). When the mold thickness is 0.5 mm, the gap in the folded structure is the smallest; thus, as the pressure increases, the rapid closure of the gap leads to a sharp decrease in the resistance of the tactile perception unit and a steep curve (with a maximum sensitivity of 11.2 kPa^−1^). As the pressure increases, after the folding angle is closed, the porous structure of the graphene electrode begins to compress, and the resistance enters a stage of slow change. However, the 0.5 mm‐thick F‐BS is too thin, causing the graphene electrode to be quickly compressed to its limit; thus, the curve rises slowly and finally maintains the maximum rate of change. In contrast, when the mold thickness is 0.9 mm, the gap in the folded structure is the largest; thus, as the pressure increases, the gap closes slowly, resulting in a gradual decrease in the resistance of the contact unit and a gentle curve. When the pressure continues to increase, the thickness change of Ecoflex and F‐BS becomes increasingly negligible, causing the resistance change of F‐BS to become increasingly gradual and lacking high sensitivity. Notably, when the mold thickness is 0.7 mm, the gap thickness of the folded structure in F‐BS is moderate, endowing it with two characteristics: high sensitivity and a large measurement range. As the pressure increases, the closure of the folded gap in F‐BS results in a highly sensitive resistance change (with a sensitivity of 6.4 kPa^−1^). When the pressure continues to increase, the compression of the graphene electrode leads to the formation of more microcurrent paths, further reducing the resistance of F‐BS and resulting in a linear and gentle change trend. To simultaneously achieve the dual advantages of high sensitivity and a large measurement range, the 0.7 mm‐thick F‐BS was adopted as the optimal parameter for the gripping robot sensor, and the resistance curve of F‐BS as a function of pressure is also presented in Figure S7. The tactile sensing unit exhibits a relatively high level of performance compared to recently reported devices (Figure S10a and Table S1). The resistance change curves of the contact unit under low pressure and high pressure were extracted separately to more intuitively observe the variation law of resistance signals (Figure 3e). Under low pressure, the folded structure is in the compression process, and the resistance decreases relatively linearly with increasing pressure, corresponding to the high‐sensitivity region of the resistance curve for the 0.7 mm‐thick F‐BS in Figure 3d. Under high pressure, the folded structure is completely closed and the graphene structure is compressed, the resistance exhibits two stages (a rapid decrease followed by a gentle decrease) as the pressure increases, which is consistent with the resistance variation characteristics of the 0.7 mm‐thick F‐BS in Figure 3d. Meanwhile, the performance of TENG under different pressures rises rapidly first and then stabilizes, so it can also be used as a pressure‐monitoring sensor (with a sensitivity of 16.7 kPa^−1^); however, the measurement range of this sensor is too small (Figure 3f; Figure S8).

The tactile perception unit also features an extremely fast real‐time sensing speed, with its raw resistance signal capable of responding to external pressure within 10 ms. When the external force is removed, the contact sensing unit can recover at an equally fast speed of 18 ms (Figure 3g; Table S3). In practical scenarios, an object always approaches first and then makes contact with the F‐BS, and the two sensing units of the F‐BS can record signal changes separately. As the object approaches, the non‐contact induction signal first increases, with its slope becoming increasingly steeper. Until the object touches the F‐BS, the non‐contact induction signal basically ceases to increase, while the tactile perception signal fluctuates rapidly, reflecting changes in pressure (Figure 3h). Experiments were designed where the object was placed at different distances from the F‐BS (1, 2, 3, 4, 5, 10, 30, 50, 70, 90, and 110 mm), demonstrating that the maximum detection distance of the F‐BS is 110 mm or even longer. The closer the object is to the F‐BS, the more intense the non‐contact sensing signal becomes, while the tactile perception signal remains completely unchanged (Figure 3i). Non‐contact sensing signals of additional materials are presented in Figure S9. The device exhibits relatively high levels of both area‐normalized performance and detection distance compared to recently reported studies (Figure S10b and Table S2). Notably, based on the triboelectric effect, the non‐contact induction unit possesses a unique function of identifying material types, with distinct differences in electrical signals corresponding to different materials (Figure 3j; Figure S11). However, the tactile perception unit does not provide feedback related to material types. The F‐BS fabricated via advanced processing methods exhibits excellent durability. After up to 10 000 mechanical contact cycles, neither of the two functional units shows any damage, and their signals remain stable (Figure 3k; Figure S12).

### 2.3 Application of F‐BS in Object Approach Sensing and Shape Detection

**FIGURE 4:**
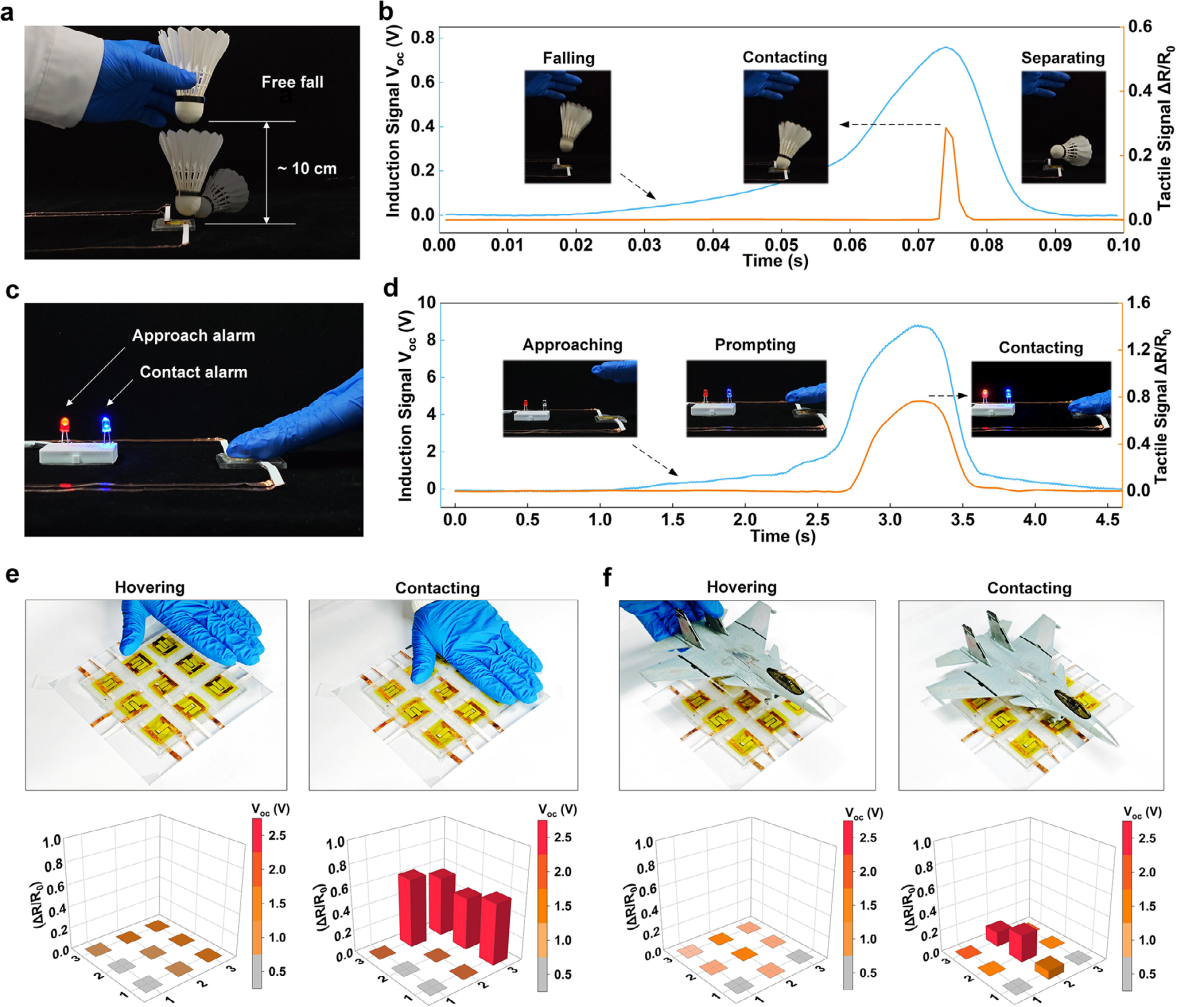
The application of F‐BS in object motion monitoring and shape sensing. (a,b) Real‐time signal changes of F‐BS at different heights during the falling process of a badminton. (c,d) Real‐time signal changes and light feedback of F‐BS when detecting finger position and pressure. (e) F‐BS arranged in an array for recognizing the hovering position of a palm and sensing pressure distribution during pressing. (f) F‐BS array for recognizing the hovering position of an aircraft model (special shape) and sensing its pressure distribution.

The performance of the F‐BS has been comprehensively optimized, and the next step is to verify its functionality. First, the F‐BS was mounted on a flat plate to detect the falling process of a badminton (Figure 4a). When the badminton falls above the F‐BS, the non‐contact induction unit promptly generates a feedback signal. Immediately afterward, the badminton collides with the F‐BS, and the signals of both the non‐contact induction unit and the tactile perception unit reach their maximum values simultaneously, then decrease as the badminton bounces away (Figure 4b; Video S1). This unique working principle is further demonstrated by the volunteer's finger interaction and the light warning system (Figure 4c). When the volunteer's finger approaches the F‐BS, the non‐contact induction unit promptly generates a feedback voltage signal. As the finger gets closer, the voltage signal increases until it exceeds the threshold to turn on the triode, activating the blue warning light. When the finger touches the F‐BS, the resistance of the tactile perception unit decreases, and the voltage divider circuit activates the red warning light (Figure 4d; Video S2).

Similarly, nine F‐BSs were fabricated into a 3 × 3 array, which is used to locate the position and shape of objects suspended in the air, as well as the pressure distribution after contact (Figure S13). When a palm is suspended in mid‐air, the color blocks on the upper and right sides of the F‐BS array change color (with increased voltage). After the palm touches the F‐BS array, these color blocks become more vivid, and at the same time, the tactile perception units feedback the changes and distribution of pressure (Figure 4e). When an aircraft model and other complex‐shaped objects are suspended, the corresponding color blocks in the F‐BS array also change color. After the aircraft model lands, the color and intensity of the color blocks real‐time reflect the approximate shape of the aircraft and the pressure distribution (Figure 4f; Figure S14).

### 2.4 Intelligent Grasping Robot Control System (IGRCS) Based on F‐BS

**FIGURE 5:**
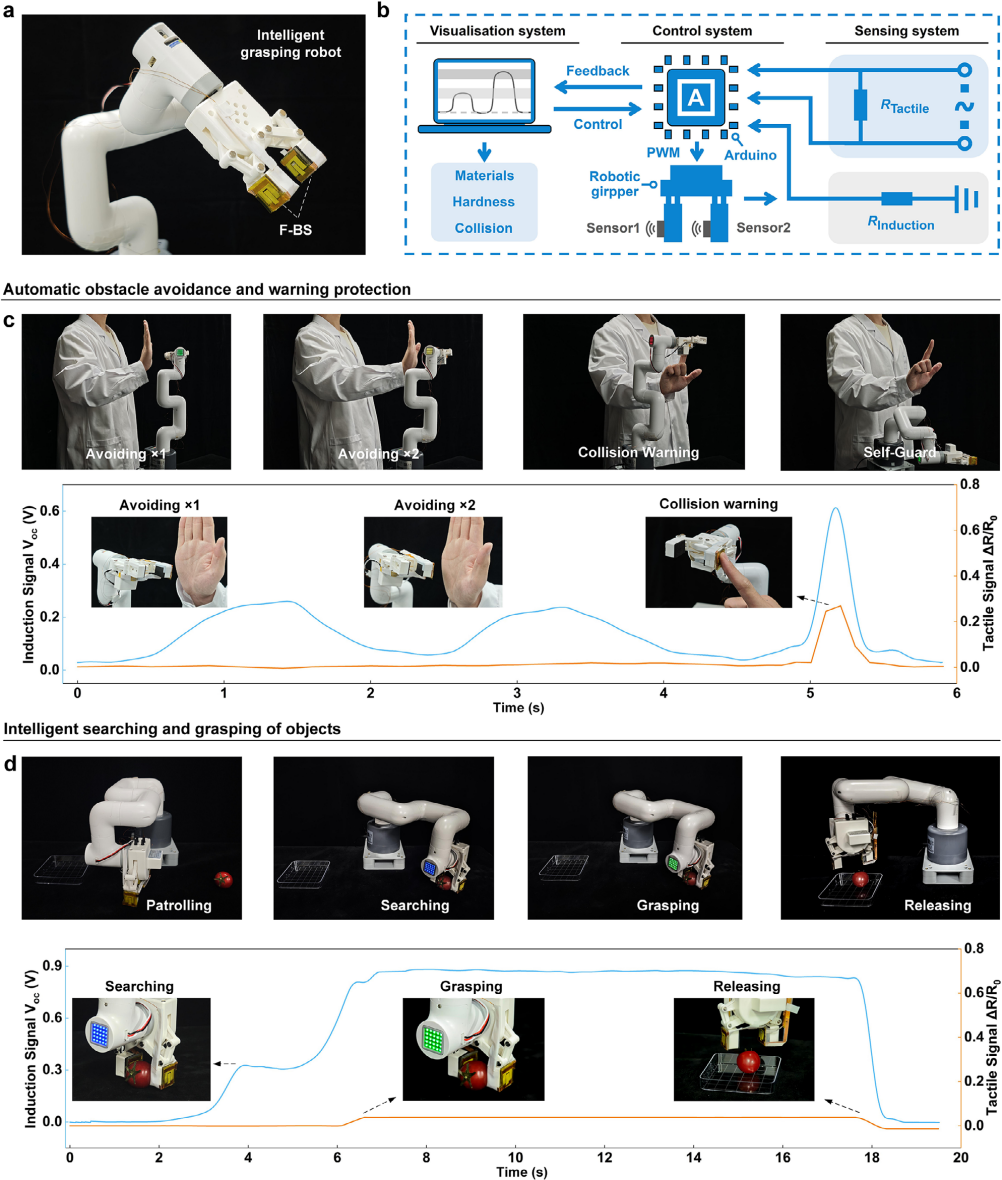
Components, obstacle avoidance, and grasping applications of the IGRCS. (a) Non‐destructive installation of F‐BSs on the inner and outer sides of the mechanical gripper. (b) Schematic diagram of the IGRCS, including the visualization system, control system, and sensing system. (c) Automatic obstacle avoidance and collision prevention of the IGR. (d) Autonomous object searching, grasping, and releasing of the IGR.

The most prominent feature of the F‐BS is its ability to simultaneously perform non‐destructive detection of two types of signals: non‐contact induction signals and contact pressure signals. This powerful function enables it to expand the perception boundary of intelligent robots. In particular, it can be non‐destructively mounted on the gripper of a robotic arm to form an intelligent grasping robot control system (IGRCS) with multi‐state perception and complex motion capabilities (Figure 5a). The IGRCS is designed to consist of three components: visualization system, control system, and sensing system. It is capable of performing material identification, hardness detection, obstacle avoidance, and vision‐denied intelligent grasping (Figure 5b). Sensor signals are transmitted in real time to the control system, where they are processed for decision‐making. The control system then provides feedback to coordinate the robotic manipulator and the visualization system accordingly. The data acquisition card operates at a sampling rate of 1000 Hz, and the overall system latency from signal transmission to decision‐making is only 90 ms, which has no adverse impact on the practical operation tasks. First, the automatic obstacle avoidance and collision early warning functions of the IGRCS were demonstrated (Figure 5c). When a volunteer's hand approached the mechanical gripper twice, the Intelligent Grasping Robot (IGR) could recognize the danger signal and perform an avoidance action on both occasions (Figure S15 and Video S3). Importantly, when a volunteer or an external object forcibly interferes with or squeezes the intelligent robot, it will immediately curl up to protect itself. The real‐time signals in Figure 5c reflect the timing of perception and response of IGR. Another important function of the IGR is automatic object grasping. While ordinary sensors alone cannot accurately search for objects or determine the optimal grasping timing, the F‐BS, with its excellent non‐contact and tactile sensing capabilities, can easily detect the position of an object and perform precise grasping (Figure 5d). When the IGR passes by an object (e.g., a small tomato), the F‐BS detects the object via its non‐contact induction unit. Immediately afterward, the control system controls the robot to perform grasping. The tactile perception unit of the F‐BS can determine the success of object grasping and release, and this high‐difficulty action can be easily achieved by relying on the powerful bimodal sensor.

**FIGURE 6:**
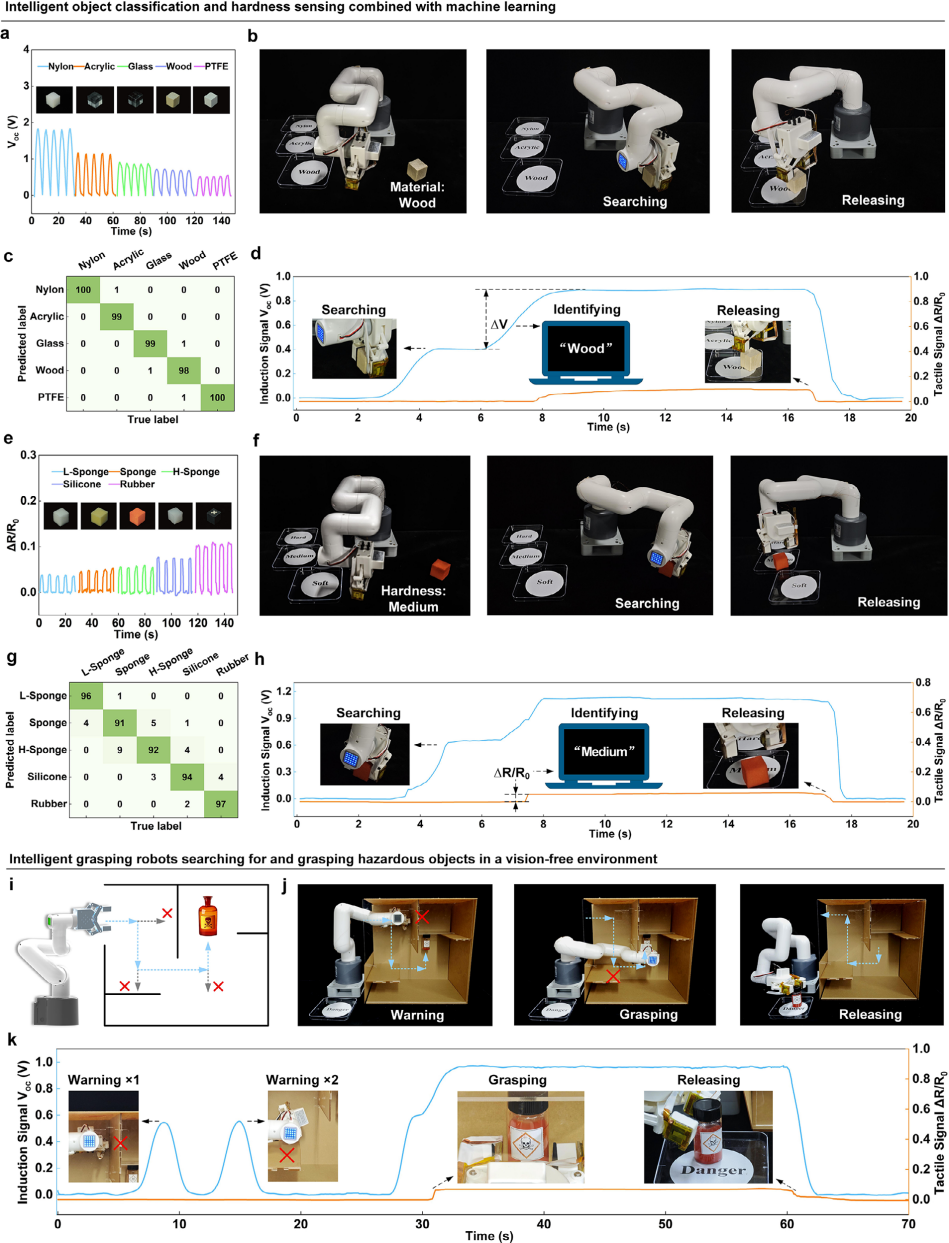
Demonstration of material detection, hardness sensing, and vision‐denied rescue operations for IGR via machine learning integration. (a) Voltage signals during the grasping of different materials. (b) Grasping, identification, and placement of wood at a specific position. (c) Material recognition success rate of the machine learning system. (d) Real‐time signals during the grasping, identification, and release of wood. (e) Voltage signals during the grasping of objects with different hardness levels. (f) Grasping, identification, and placement of H‐Sponge at a specific position. (g) Hardness recognition success rate of the machine learning system. (h) Real‐time signals during the grasping, identification, and release of H‐Sponge. (i) Schematic route of IGR operation in a vision‐denied environment. (j,k) Obstacle avoidance, hazardous object search, and grasping by the IGR in a vision‐denied environment.

Relying on the non‐contact induction unit of the F‐BS, the IGR can easily identify different material types through the voltage signals fed back during the grasping action of the gripper, such as nylon, acrylic, glass, wood, PTFE, etc. (Figure 6a). Taking wood as the target object, the IGR determines the material type based on the voltage variation, displays the judgment result via the visualization system, and places it in the corresponding area (Figure 6b,d; Video S4). Real‐time pressure and distance signals are displayed in Figure S16, while the identification and classification of other materials are provided in Figure S17. Machine learning is used to train and memorize the voltage signal features of different materials, and assist the IGR in conducting a new round of feature recognition. After training with 500 sets of sample data for each material and 100 times of feature recognition, the accuracy rate of material type recognition exceeds 99% (Figure 6c).

Similarly, the tactile perception unit of the F‐BS is highly sensitive to pressure and is used to identify the hardness of different materials, such as low‐density sponge (L‐Sponge), sponge, high‐density sponge (H‐Sponge), silica gel, rubber, etc. (Figure 6e). When the target object is an H‐Sponge, the IGR judges the material hardness based on the resistance change rate, places it in the corresponding area, and feeds the judgment result back to the visualization system (Figure 6f,h; Video S5). Real‐time pressure and distance signals in the hardness recognition experiment are shown in Figure S18, and the discrimination and classification of objects with different hardness levels are presented in Figure S19. With the assistance of machine learning, the average accuracy rate of hardness recognition exceeds 94% (Figure 6g). This accuracy achieves a level consistent with advanced sensors reported in the field.

Owing to the unique capabilities of the F‐BS, it will be innovatively applied to disaster sites and hazardous operations in vision‐denied spaces. A fully enclosed complex vision‐free maze was constructed, and hazardous drugs were placed at the end of the maze. The IGR needs to navigate through multiple obstacles to locate the hazardous objects and retrieve them (Figure 6i; Figure S20). When the IGR travels to the front of the first wall, the F‐BS feeds back an early warning signal, and the control system immediately changes the traveling direction of the IGR. Similarly, the IGR also bypasses the second wall and reaches an open area. Through the sweeping movement of the gripper, the IGR locates the hazardous object and grips it to a safe area (Figure 6j,k; Video S6). The entire process of obstacle avoidance, searching, grasping, and releasing mentioned above is carried out without human intervention. In summary, the IGRCS can quickly and accurately complete grasping tasks in vision‐denied spaces without relying on a vision system, which will provide new ideas for fields such as natural disaster rescue, high‐risk operations, and advanced human‐machine interface.

## 3 Conclusion

This study proposes a folded bimodal sensor (F‐BS) based on a monolithic laser‑induced graphene film through an elegant substrate folding design. By integrating the triboelectric nanogenerator and graphene piezoresistive effect, the F‐BS achieves rapid programmable fabrication, a simple manufacturing process, and a high level of integration. Two core functions, namely non‑contact induction and tactile perception, are seamlessly combined within a single device, enabling long‑range target detection of up to 110 mm and highly sensitive pressure sensing with a maximum sensitivity of 11.2 kPa^−^^1^. The F‐BS is nondestructively integrated onto a robotic gripper, forming an anthropomorphic intelligent grasping system with superior multimodal sensing capabilities. By leveraging machine learning to fuse sensory information from various objects, the system accurately identifies material and hardness with a recognition accuracy of up to 99%. Notably, it can precisely locate hazardous objects or injured individuals for rescue operations, even in vision‑denied environments. Overall, this work provides a multi‑source sensing foundation for artificial intelligence hardware, empowering robots to perceive spatial position, intelligently recognize objects, and perform grasping interactions. It also establishes a promising interaction interface between artificial intelligence and advanced robotic systems.

## 4 Experimental Section

### 4.1 Fabrication of the F‐BS

A laser platform (DLS 2.3, Universal Laser Systems, Inc.) equipped with a CO^2^ laser with a wavelength of 10.6 µm and beam size of ≈100 µm was used for irradiating fluorine‐doped polyimide (F‐PI) film (100 µm in thickness). The 4,4'‐diamino‐2,2'‐bis (trifluoromethyl) biphenyl (6FODA) was mixed with 4,4'‐oxydianiline (ODA) at different mass fractions (20, 40, 60, 80, 100 wt.%), and then blended with pyromellitic dianhydride (PMDA) of equal mass in N,N‐dimethylacetamide (DMAc), a polar aprotic reaction solvent. The mixture was stirred at room temperature for 5 h to ensure complete reaction. The solution was coated onto a clean glass mold, which was then placed in a vacuum oven at 80°C for 3 h. After the solvent evaporated, the thermal imidization process was completed by maintaining temperatures at 100°C, 200°C, and 250°C for 50 min each (heating rate: 2°C/min). Finally, the mold was fully immersed in deionized water to peel off the F‐PI film. A laser with a power of 1.25 W was used to fabricate graphene electrodes (Non‐contact induction unit area: 10 × 10 mm^2^, tactile perception unit area: 10 × 5 mm^2^). After folding, Ecoflex (Smooth‐on Ecoflex 00–30) was cast to form a single F‐BS unit (area: ∼20 × 20 mm^2^). The F‐BS array was assembled by bonding nine individual units into an integrated structure via Ecoflex curing.

### 4.2 Electrical Measurement

The excitation platform was a linear motor (B01‐37×120/160, LinMot). The voltage of the F‐BS is measured by a spectrum analyzer (MDO3034, Tektronix, USA), and the capacitance voltage was tested by a digital multimeter (7510, Keithley, USA). The pressure sensing range of TENG was evaluated using an E44.104 tensile machine (10 kN load cell, MTS Systems Corp.). SEM was performed with JEOL JSM 7001F at 10 kV to examine the morphologies of the laser scribed features. A Horiba HR800 Raman microscope was employed to obtain Raman spectra with a 532 nm excitation laser at the power of 5 mW. XRD characterization was performed on Rigaku D/max 2550 with Cu Kα radiation (λ = 1.54°). XPS (Thermo Fisher ESCALAB 250Xi) was applied for comparing the content of surface elements. Prepared by dropping of LIG‐alcohol suspension on a Cu grid, TEM images were taken using an 80 keV JEM 2100F (JEOL Inc.) for evaluation of LIG at nano scale.

All volunteers have known all the details about the experiment. The experiment results will be used to conduct further research. Beihang University will ensure the health and safety of all volunteers in the experiment. All volunteers have agreed to participate in the experiment.

## Author Contributions

**Weixiong Yang**: conceptualization, methodology, data curation, investigation, writing – original draft, formal analysis. **Yuhan Guo**: methodology, data curation, formal analysis. **Mingguang Han**: review and editing. **Bin Feng**: methodology, review and editing. **Yuan Ma**: methodology, review and editing. **Bingang Xu**: methodology, review and editing. **Fu Liu**: investigation, formal analysis. **Dan Wang**: methodology, formal analysis. **Pingping Hao**: funding acquisition, supervision. **Sida Luo**: conceptualization, funding acquisition, methodology, writing – original draft, formal analysis, supervision. **Xilun Ding**: conceptualization, funding acquisition, methodology, writing – original draft, formal analysis, supervision. All authors discussed and commented on the manuscript.

## Conflicts of Interest

The authors declare no conflict of interest.

## Supporting information




**Supporting File 1**: advs75868‐sup‐0001‐SuppMat.docx.

**Supporting File 2**: advs75868‐sup‐0002‐VideoS1.mov.

**Supporting File 3**: advs75868‐sup‐0003‐VideoS2.mov.

**Supporting File 4**: advs75868‐sup‐0004‐VideoS3.mov.

**Supporting File 5**: advs75868‐sup‐0005‐VideoS4.mov.

**Supporting File 6**: advs75868‐sup‐0006‐VideoS5.mov.

**Supporting File 7**: advs75868‐sup‐0007‐VideoS6.mov.

## Data Availability

The data that support the findings of this study are available from the corresponding author upon reasonable request.
