# Supplementary material for: A Folded, Structure‐Integrated Bimodal Sensor Enabling Non‐Contact and Tactile Perception for Intelligent Robots
Source: Adv Sci (Weinh). 2026 May 28:e75868. Online ahead of print. doi: 10.1002/advs.75868 (PMC13335930; doi:10.1002/advs.75868)
Supplement: Supplementary file 1 — Supporting File 1: advs75868‐sup‐0001‐SuppMat.docx. [file ADVS-9999-e75868-s007.docx]

**Supporting Information**

**A Folded, Structure-Integrated Bimodal Sensor Enabling Non-Contact and Tactile Perception for Intelligent Robots**

Weixiong Yang^1^†, Yuhan Guo^1^†, Mingguang Han^1^, Bin Feng^1^, Yuan Ma^2^, Bingang Xu^3^, Fu Liu^4^, Dan Wang^1^, Pingping Hao^1^, Xilun Ding^1^*, Sida Luo^1^*


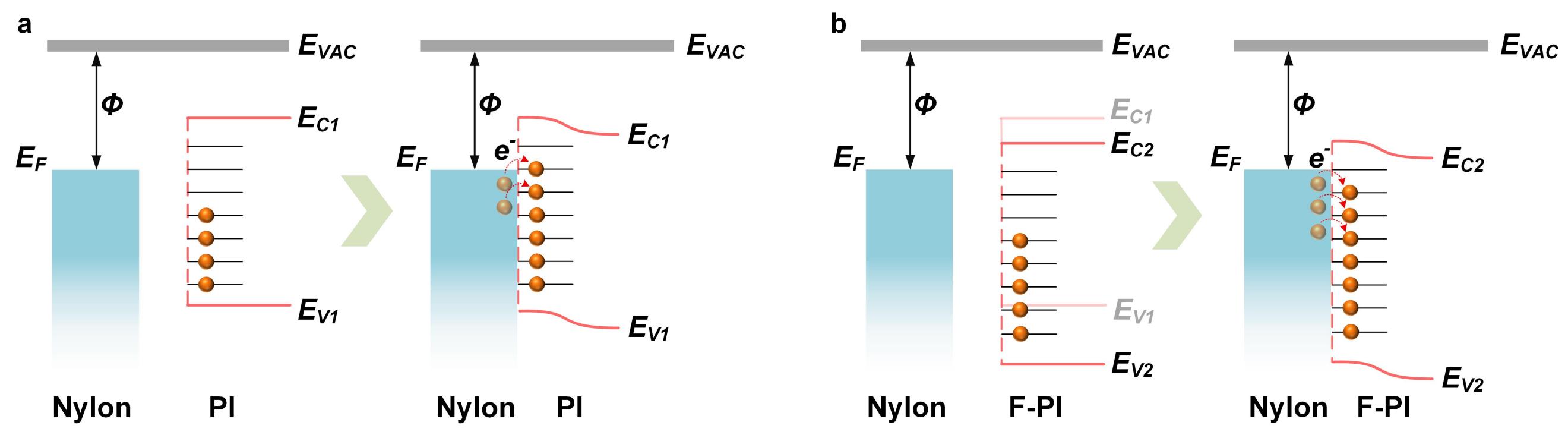


**Figure S1.** Modified surface states model. **a** Surface charge transfer in the Nylon-PI system during contact electrification. **b** Surface charge transfer in the Nylon-F-PI system during contact electrification.

*Φ* is the Nylon work function, *E_F_* is the Fermi level, *E_VAC_* is the vacuum level, *E_C1_* and *E_V1_* represent the conduction band and valence band of PI, respectively, while *E_C2_* and *E_V2_* correspond to those of F-PI. Fluorine, with its strong electronegativity, increases the electron affinity of the material, which is defined as the energy difference between the vacuum level (*E_VAC_*) and the conduction band (*E_C_*). The incorporation of fluorine enhances the electron affinity of F-PI (Figure S1b), lowering its conduction band (*E_C2_*). This allows electrons to transfer more easily to the F-PI surface during contact electrification, macroscopically manifesting as higher charge density and enhanced electrical performance of the F-PI-TENG. The introduction of fluorine also lowers the valence band of F-PI (*E_V2_*), leading to a wider energy gap (*E_V2_-E_C2_*) than that of pristine PI (*E_V1_-E_C1_*). This larger energy gap contributes to enhanced electron stability and facilitates electron transfer. Fluorine incorporation enhances the work function of the material, resulting in a lower surface potential. This indicates that fluorine enables the material to more easily accumulate negative charge.


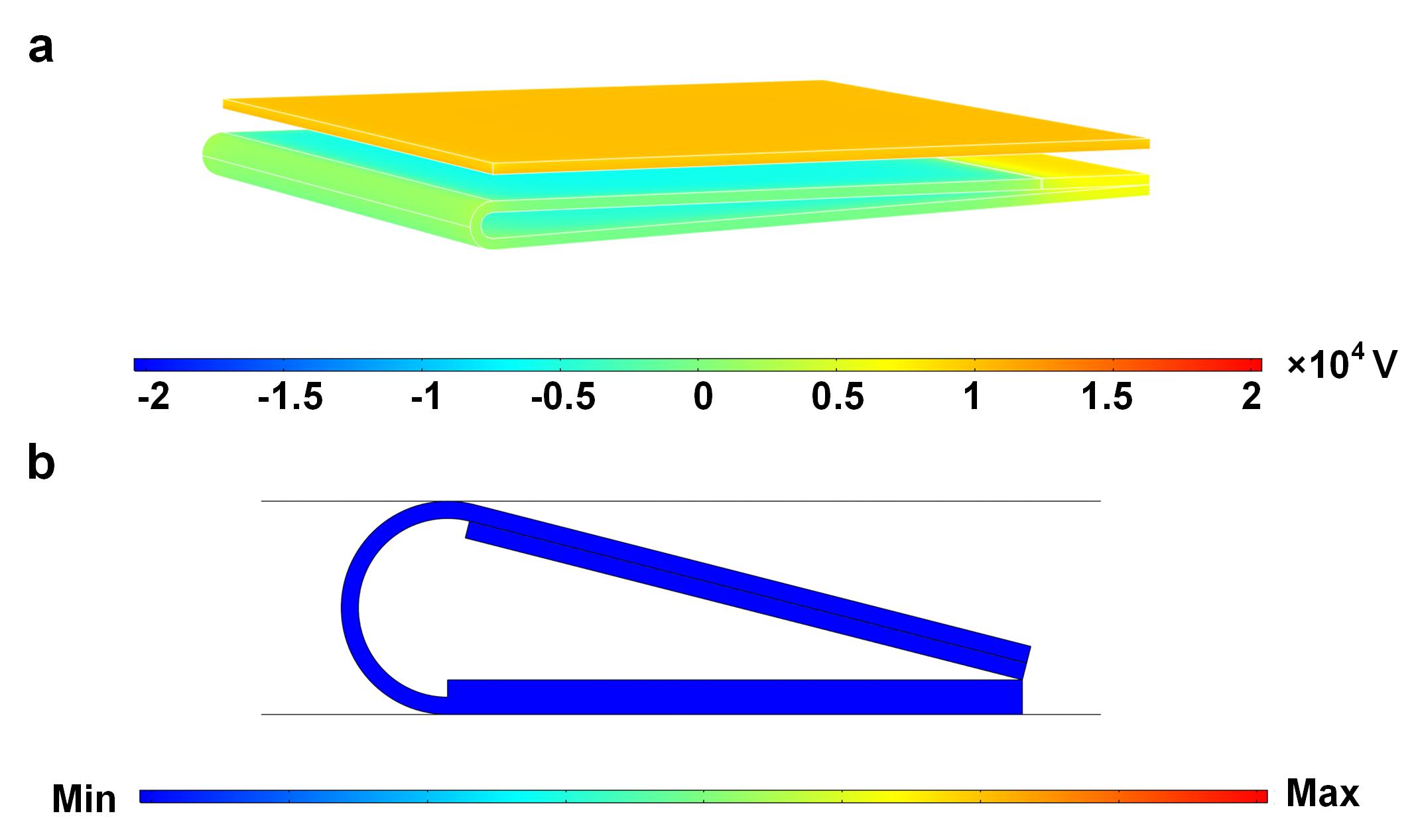


**Figure S2.** **a** The distribution state of the electric potential field when an external object is at a medium distance. **b** Mechanical simulation of the folded structure in its initial state.


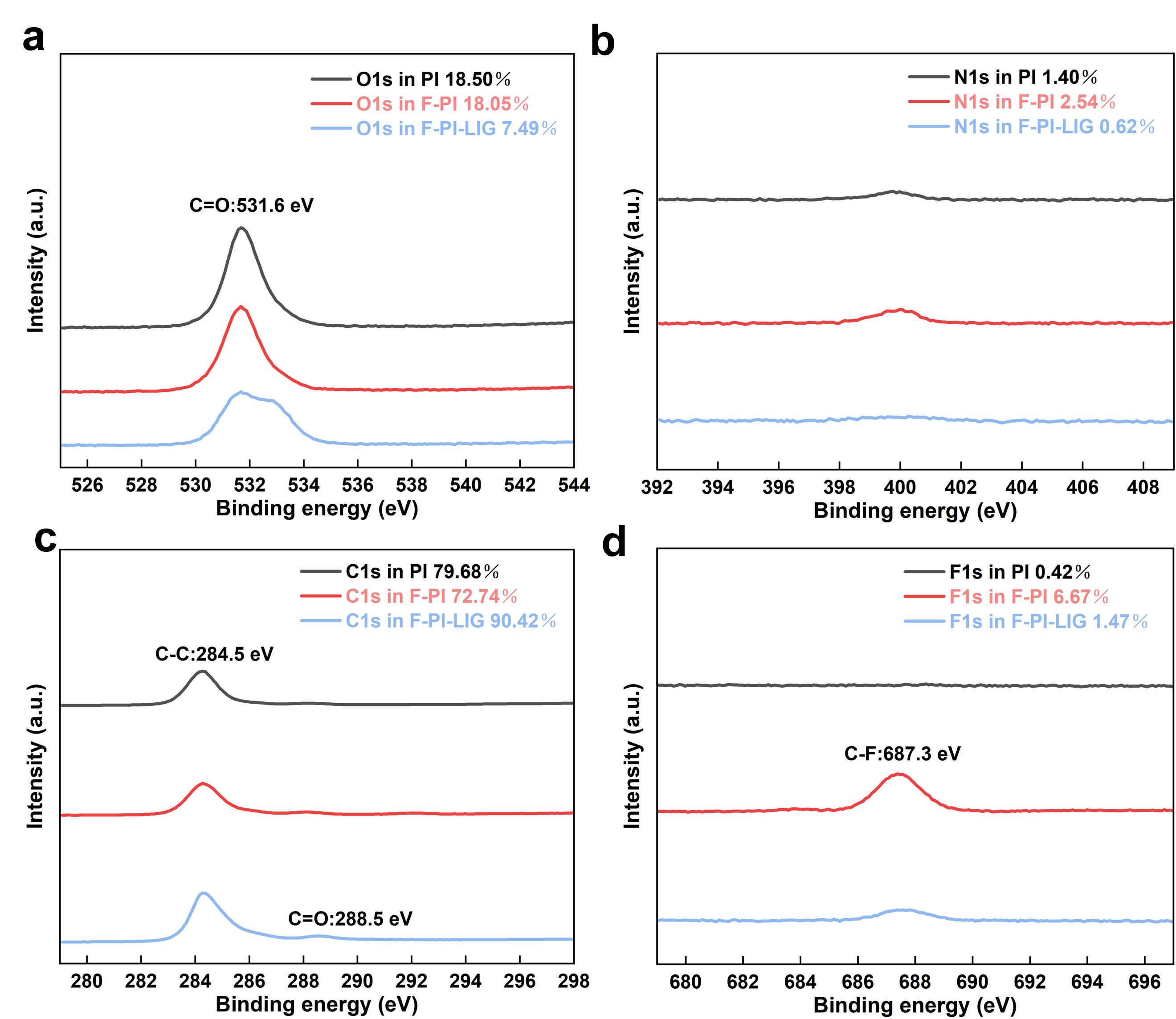


**Figure S3.** XPS pattern of **a** O1s, **b** N1s,**c** C1s and **d** F1s of the original PI, F-PI and F-PI-LIG.

High resolution O1s XPS spectrum of PI, F-PI and F-PI-LIG. High resolution N1s XPS spectrum of PI, F-PI and F-PI-LIG. High resolution C1s XPS spectrum of the PI, F-PI and F-PI-LIG, showing the dominant C-C peak. High resolution F1s XPS spectrum of PI, F-PI and F-PI-LIG. The intensity of the O1s, N1s and F1s peaks were greatly reduced after laser exposure.


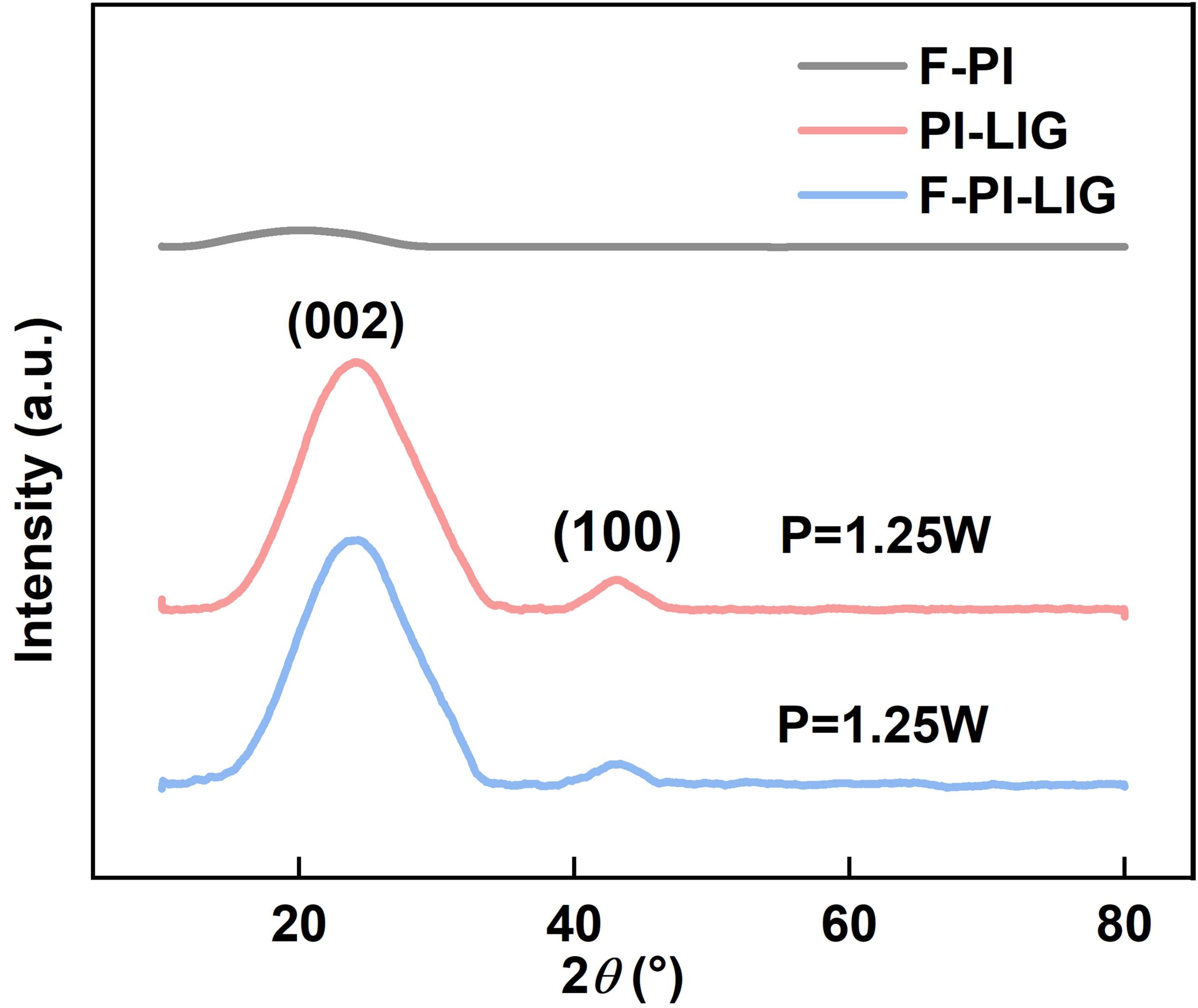


**Figure S4.** XRD pattern of the F-PI, PI-LIG and F-PI-LIG.

The XRD pattern in the supporting information confirmed the existence of multilayer internal structure of graphene, and the strong peaks (002) and (100) were concentrated at 2*θ* = 24.3°and 2*θ* = 42.9°, respectively.


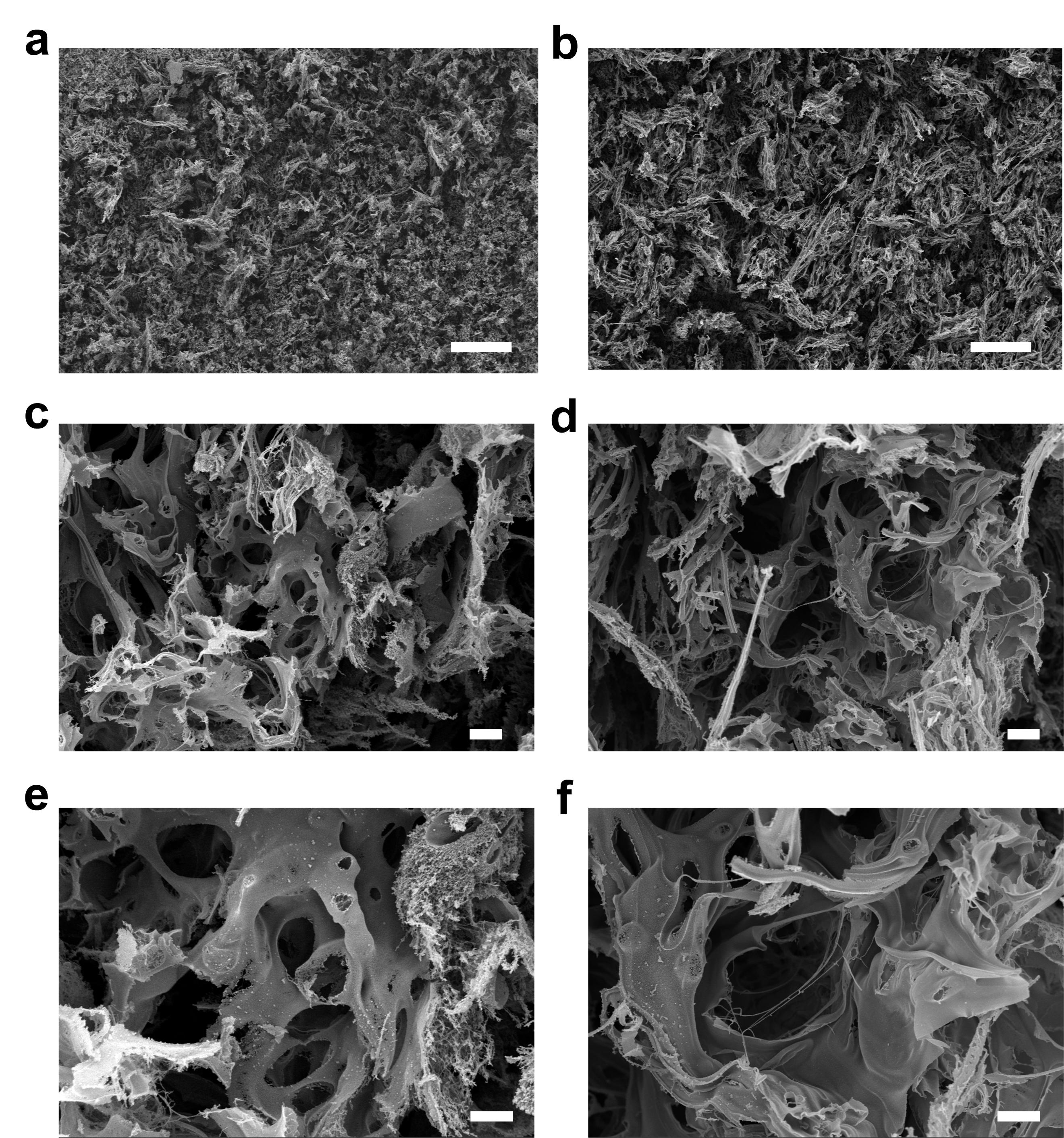


Figure S5. The SEM images under the endurance test. **a-b** Low (scale = 100 μm) magnified SEM images of PI-LIG and F-PI-LIG. **c-d** High (scale = 4 μm) magnified SEM images of PI-LIG and F-PI-LIG. **e-f** High (scale = 2 μm) magnified SEM images of PI-LIG and F-PI-LIG.


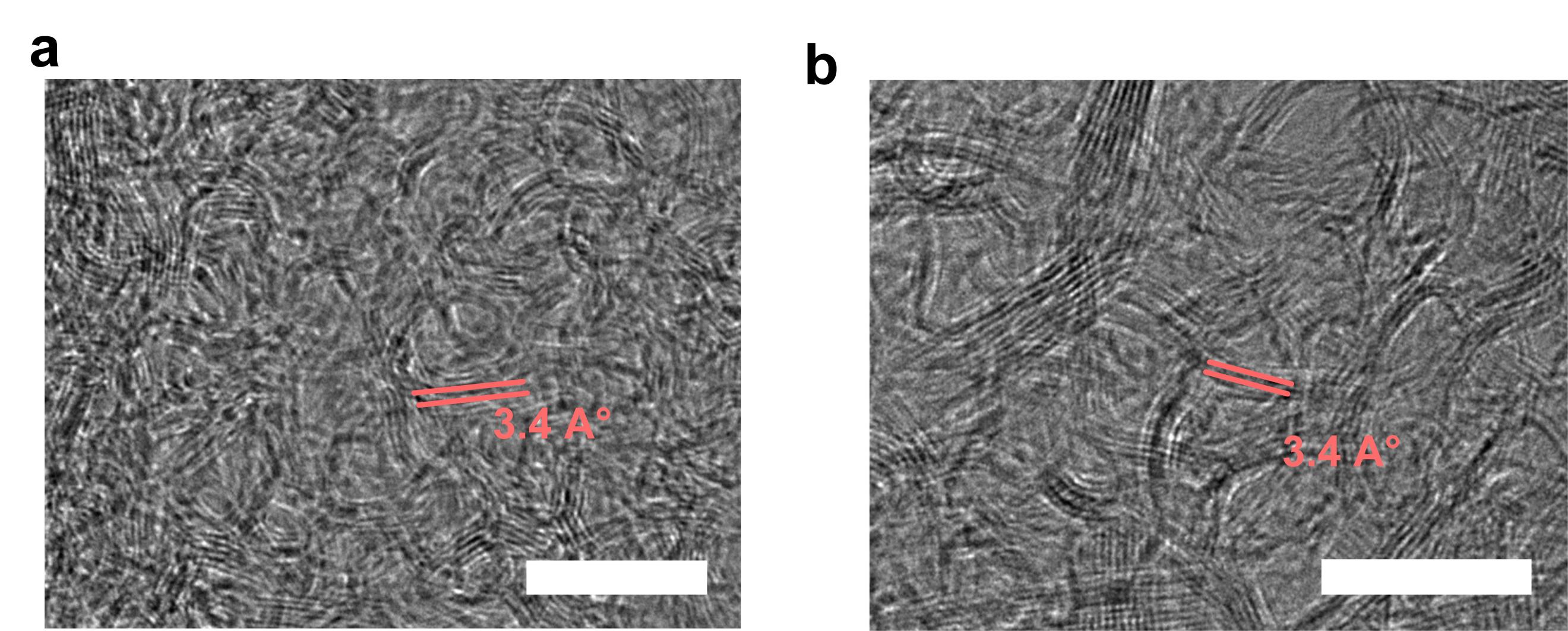


Figure S6. TEM images of PI-LIG and F-PI-LIG (scale = 10 nm).


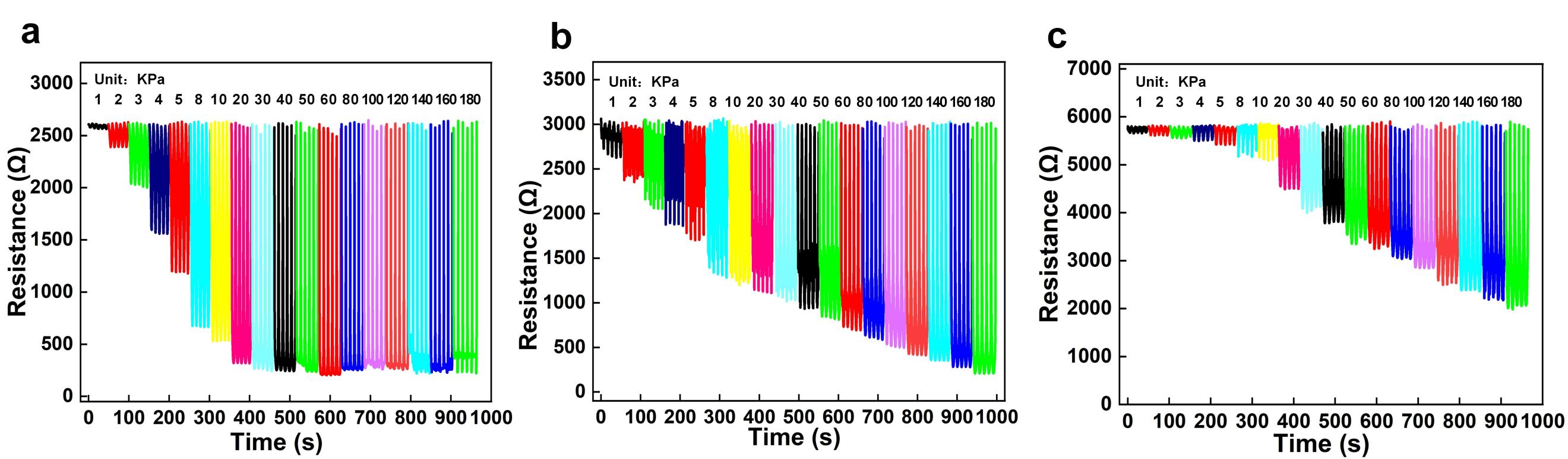


Figure S7. Tactile sensing performance under different pressures with different mold thicknesses (0.5, 0.7, 0.9 mm).


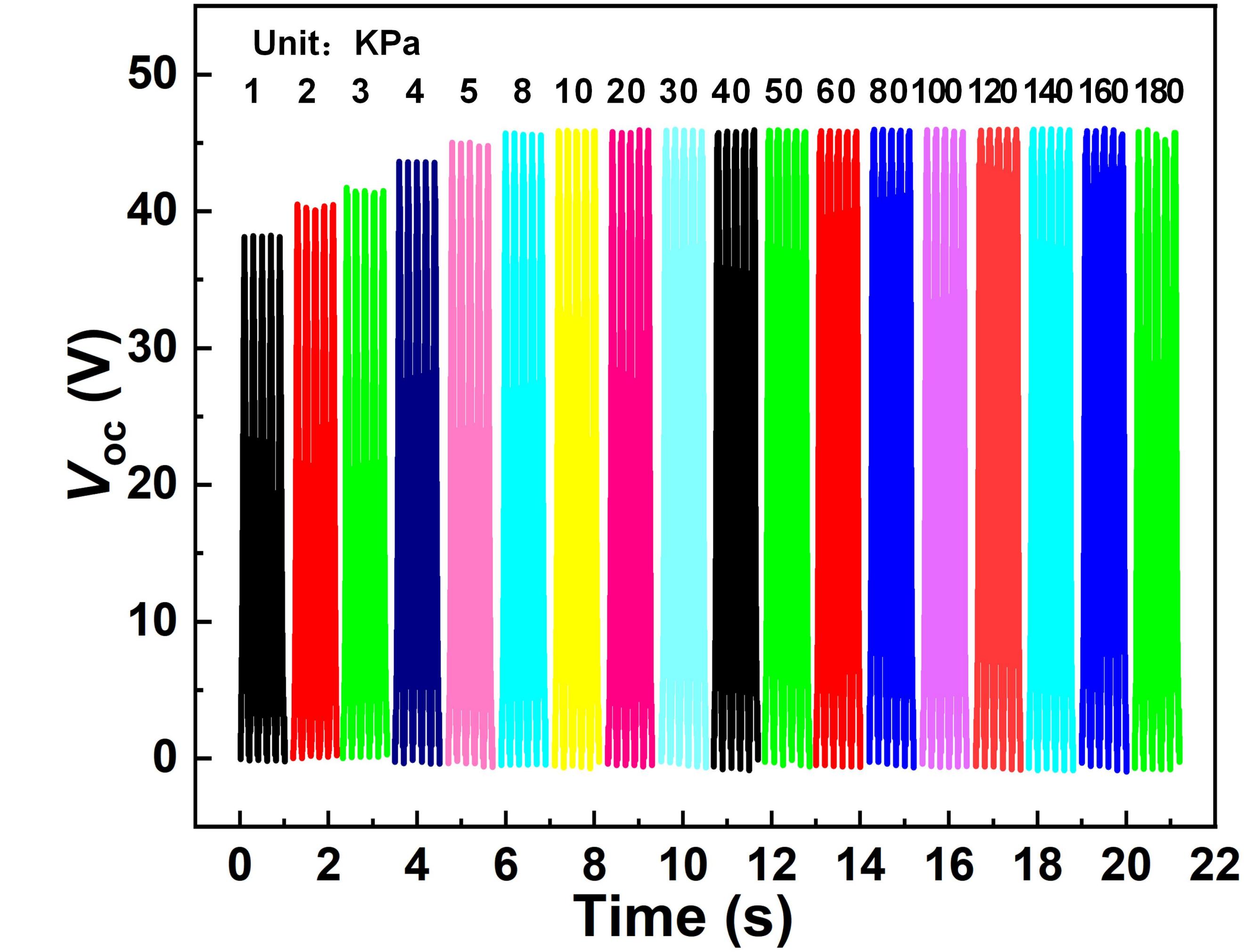


Figure S8. Voltage performance comparison of TENGs under different pressures.


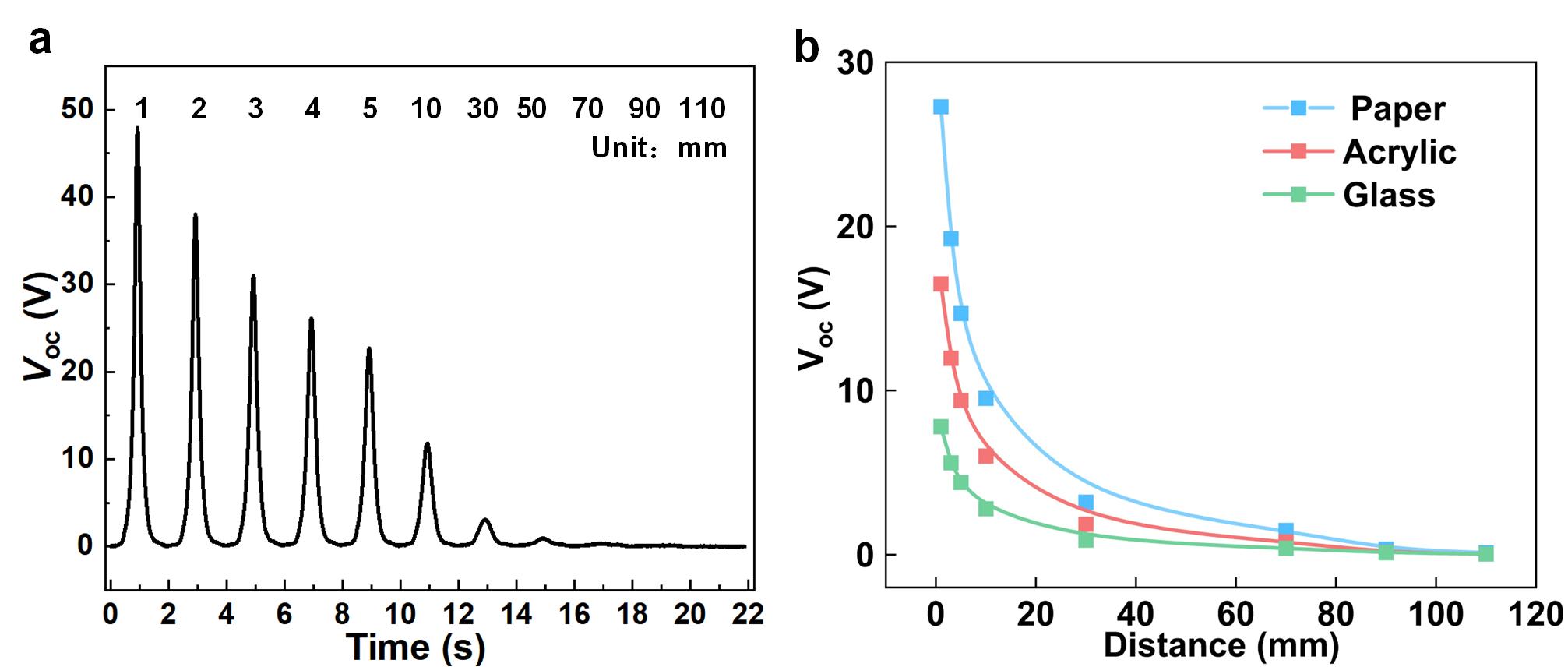


Figure S9. a Performance comparison of the non-contact induction unit at different distances. **b** Non-contact signals of other representative materials at different distances.


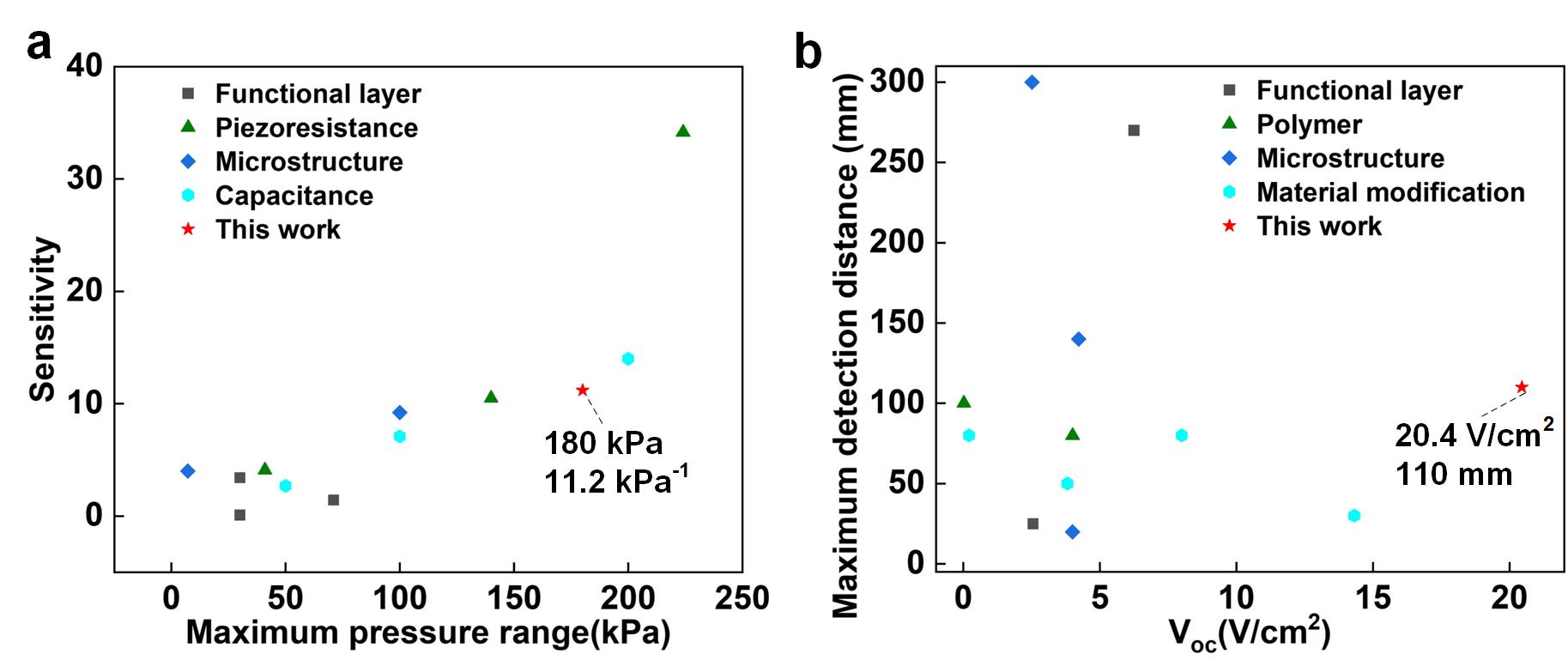


Figure S10. Performance comparison between the F-BS and recently reported studies. **a** Comparison of tactile perception sensitivity and detection range^[1–11]^. **b** Comparison of the performance and detection distance of the non-contact induction unit^[1–3,12–19]^.


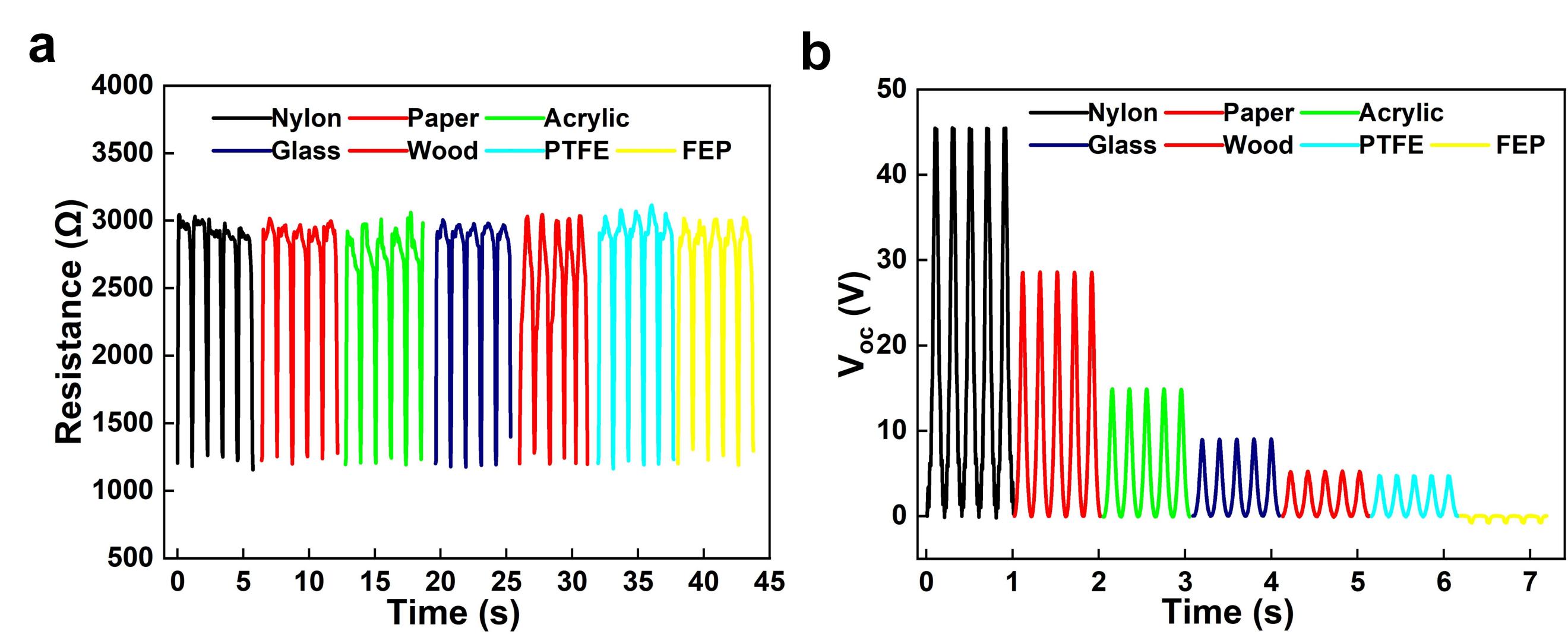


Figure S11. Signals of F-BS when in contact with different materials. **a** Performance comparison of the tactile perception unit at different materials. **b** Performance comparison of the non-contact induction unit at different materials.


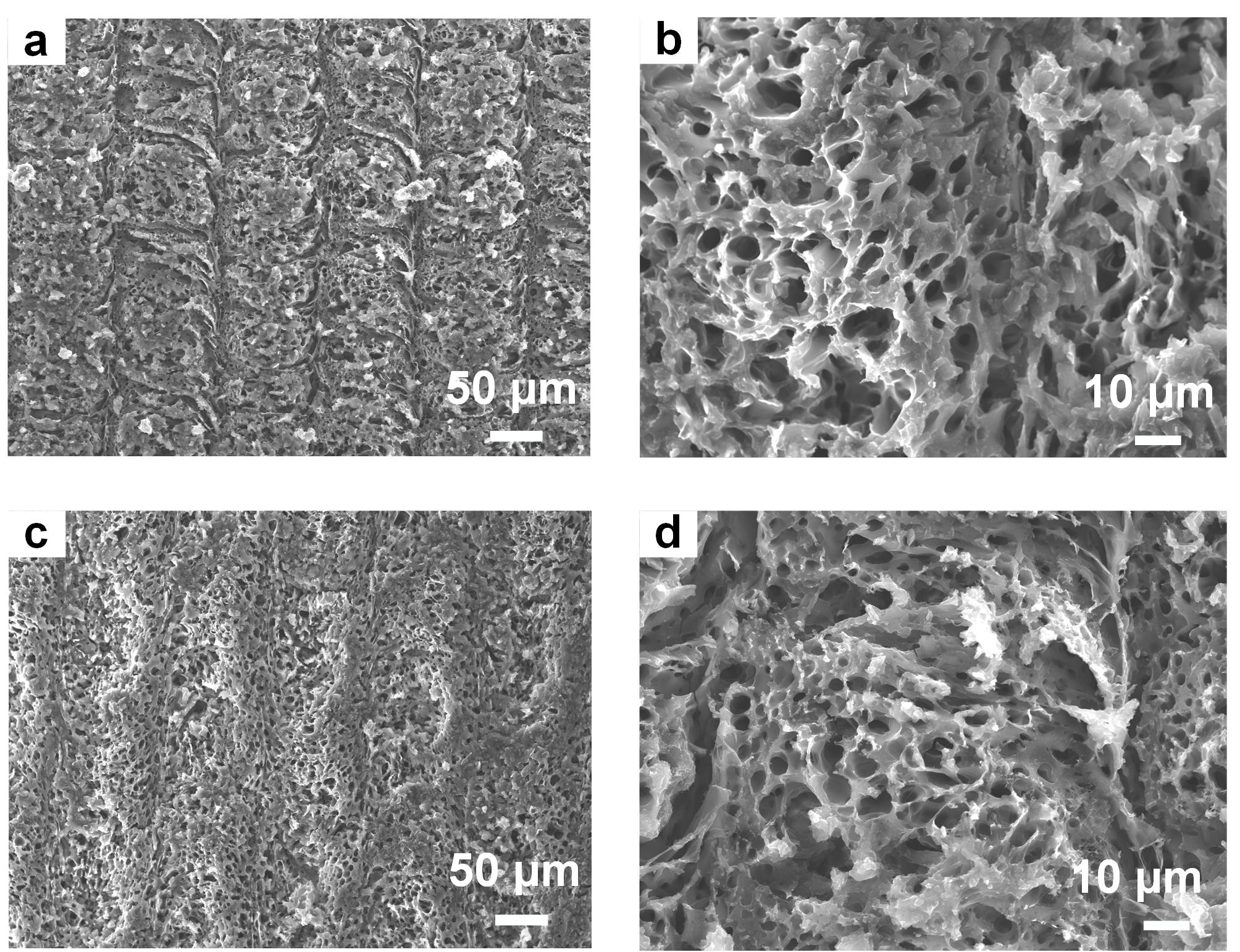


Figure S12. SEM images of the graphene electrode before the durability test at **a** low and **b** high magnification, and after the durability test at **c** low and **d** high magnification.


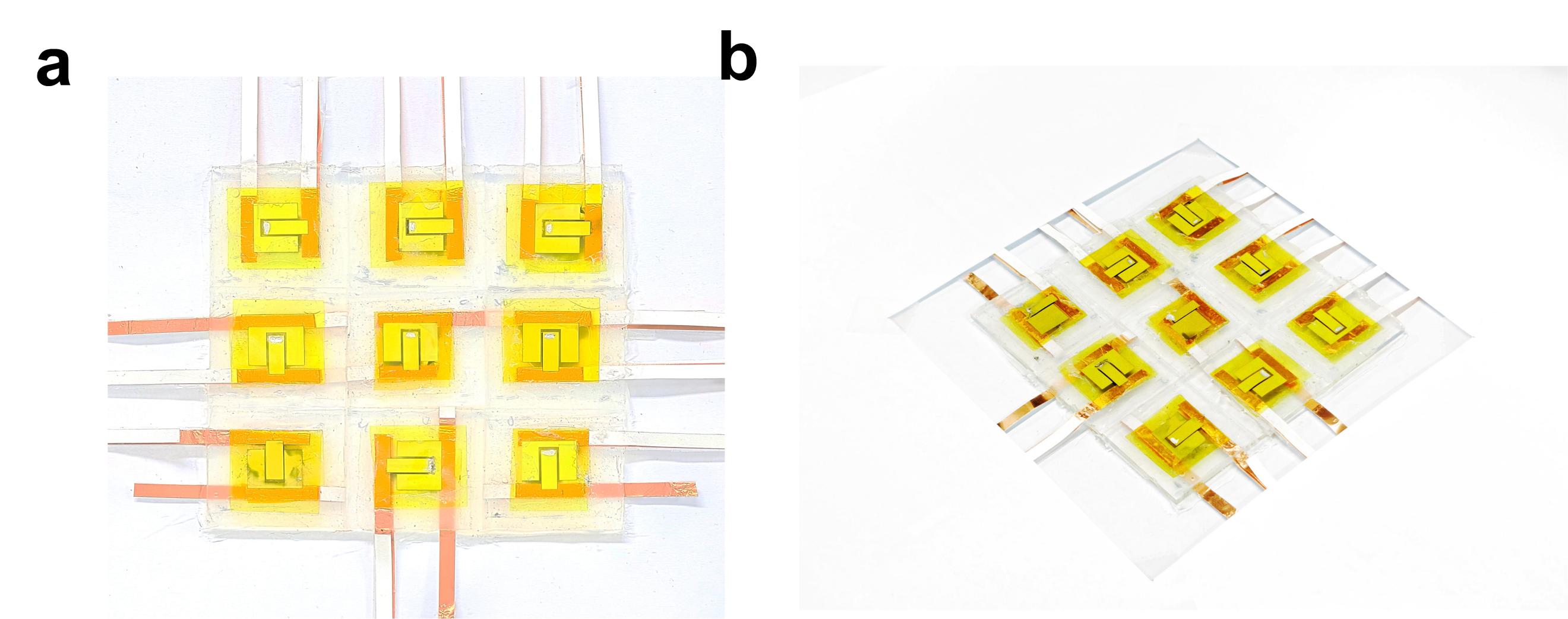


Figure S13. An array composed of arranged F-BS units.


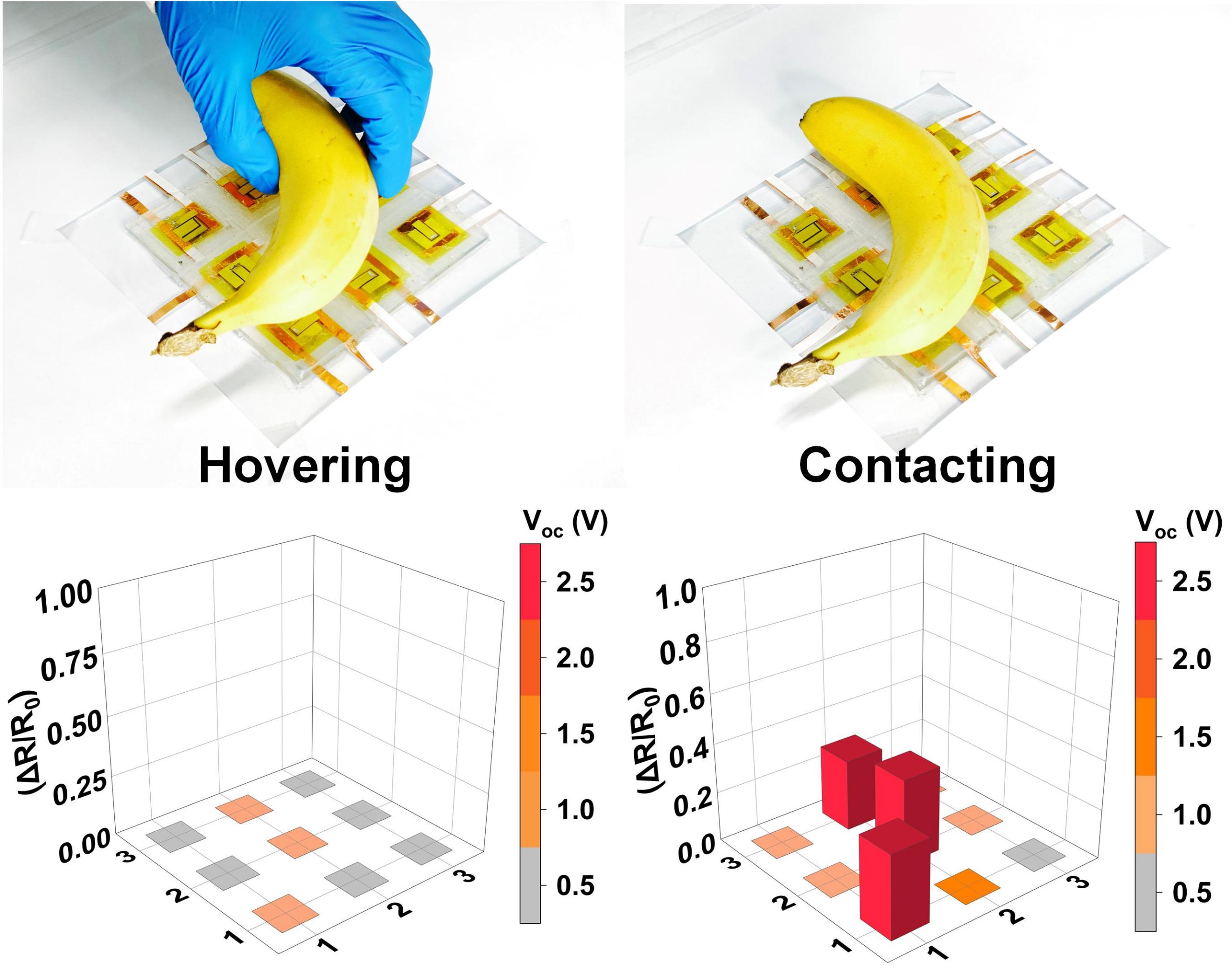


Figure S14. F-BS array for recognizing the hovering position of the banana (special shape) and sensing its pressure distribution.


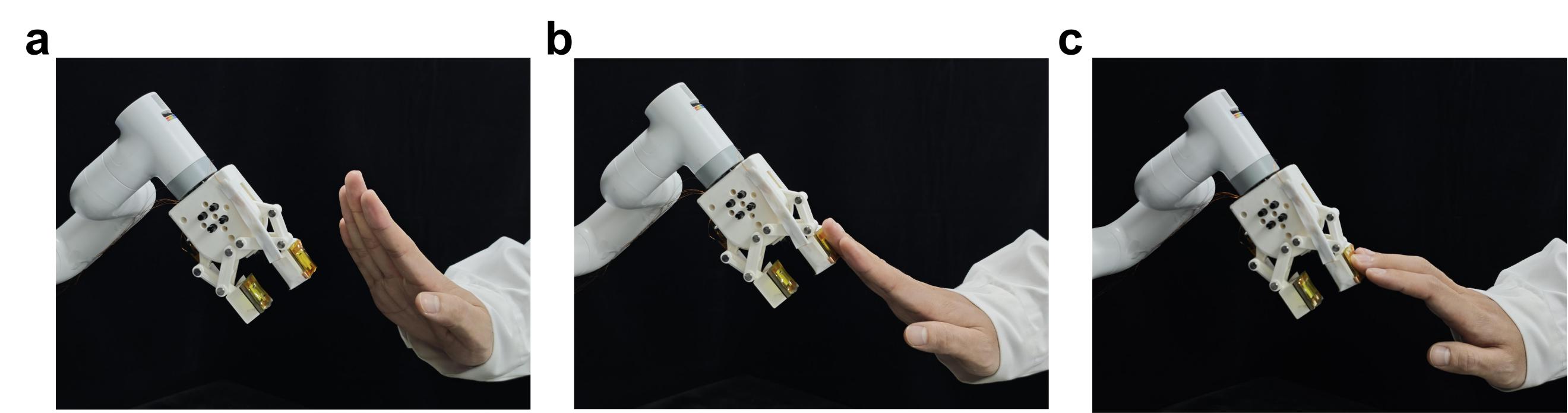


Figure S15. The F-BS monitors different positions and pressures of the hand. **a** The F-BS detects the hand approaching. **b** The F-BS senses finger contact. **c** The F-BS monitors finger pressure.


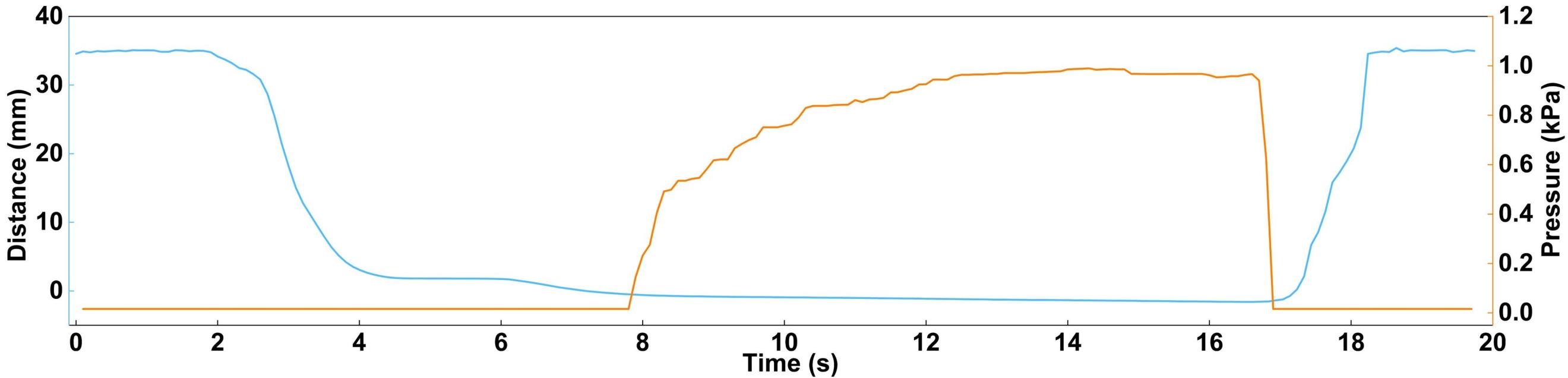


Figure S16. Real-time pressure and distance signals in material recognition.


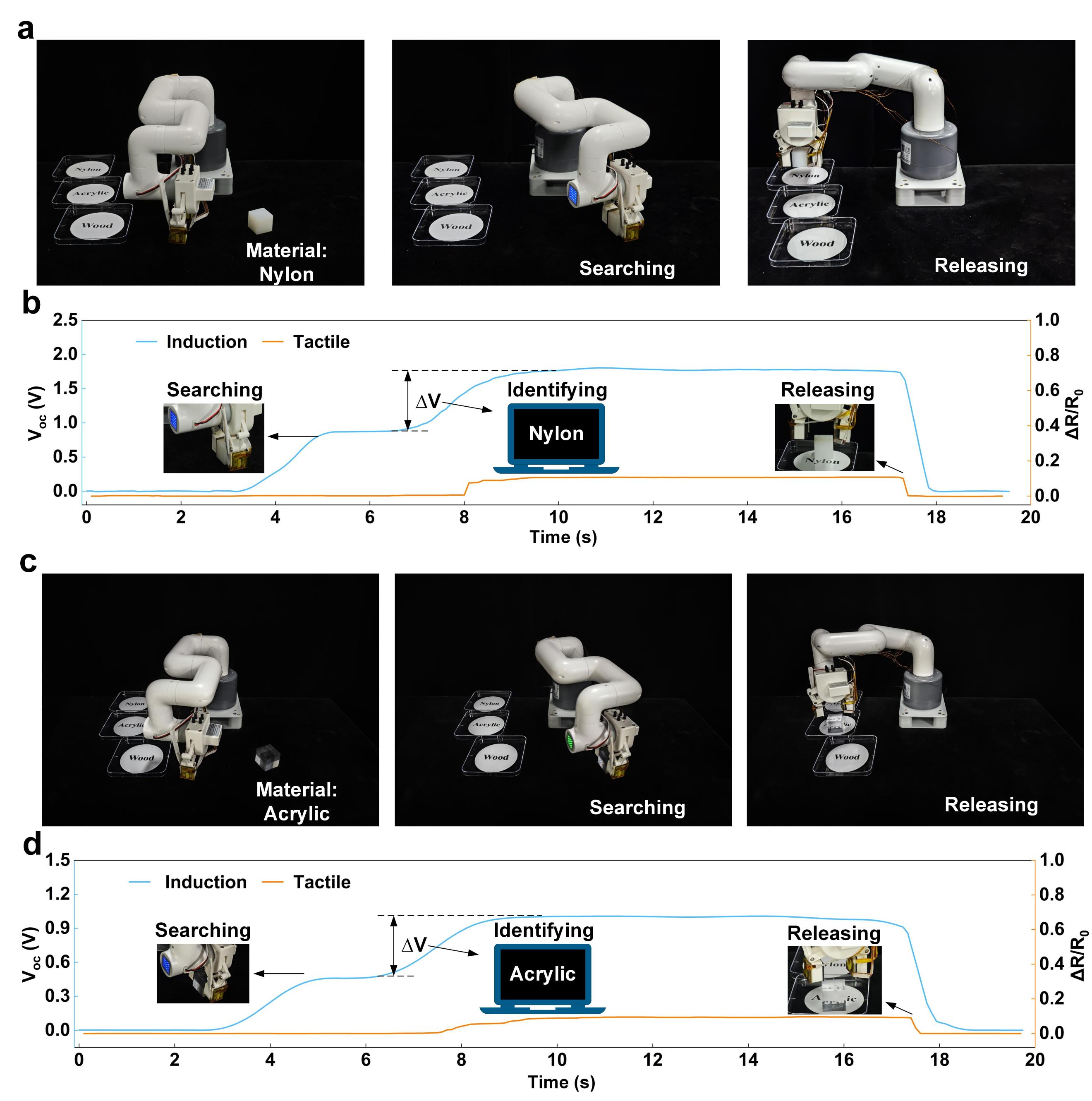


Figure S17. **a** Grasping, identification, and placement of nylon at a specific position. **b** Real-time signals during the grasping, identification, and release of nylon. **c** Grasping, identification, and placement of acrylic at a specific position. **d** Real-time signals during the grasping, identification, and release of acrylic.


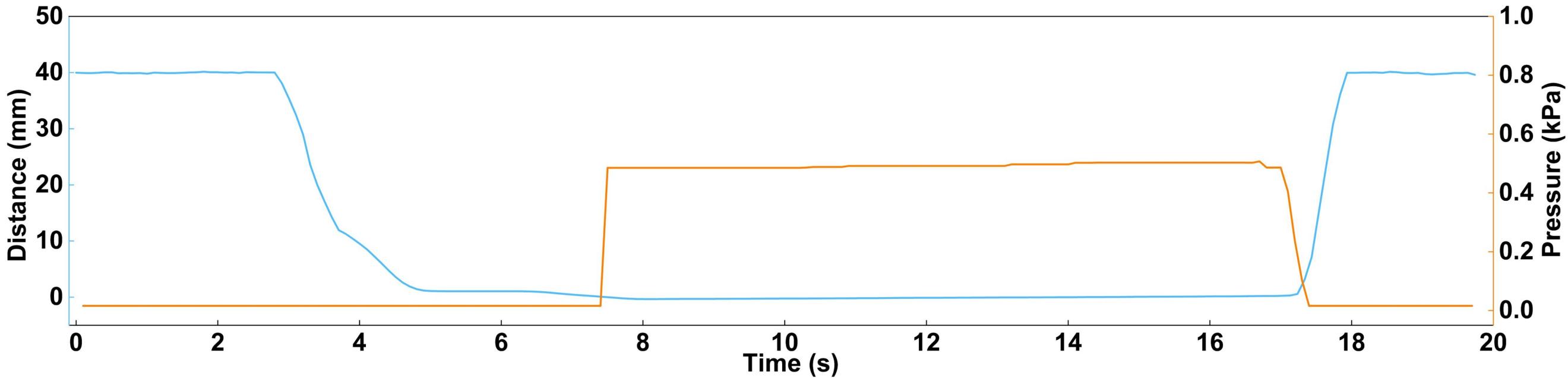


Figure S18. Real-time pressure and distance signals in the hardness recognition.


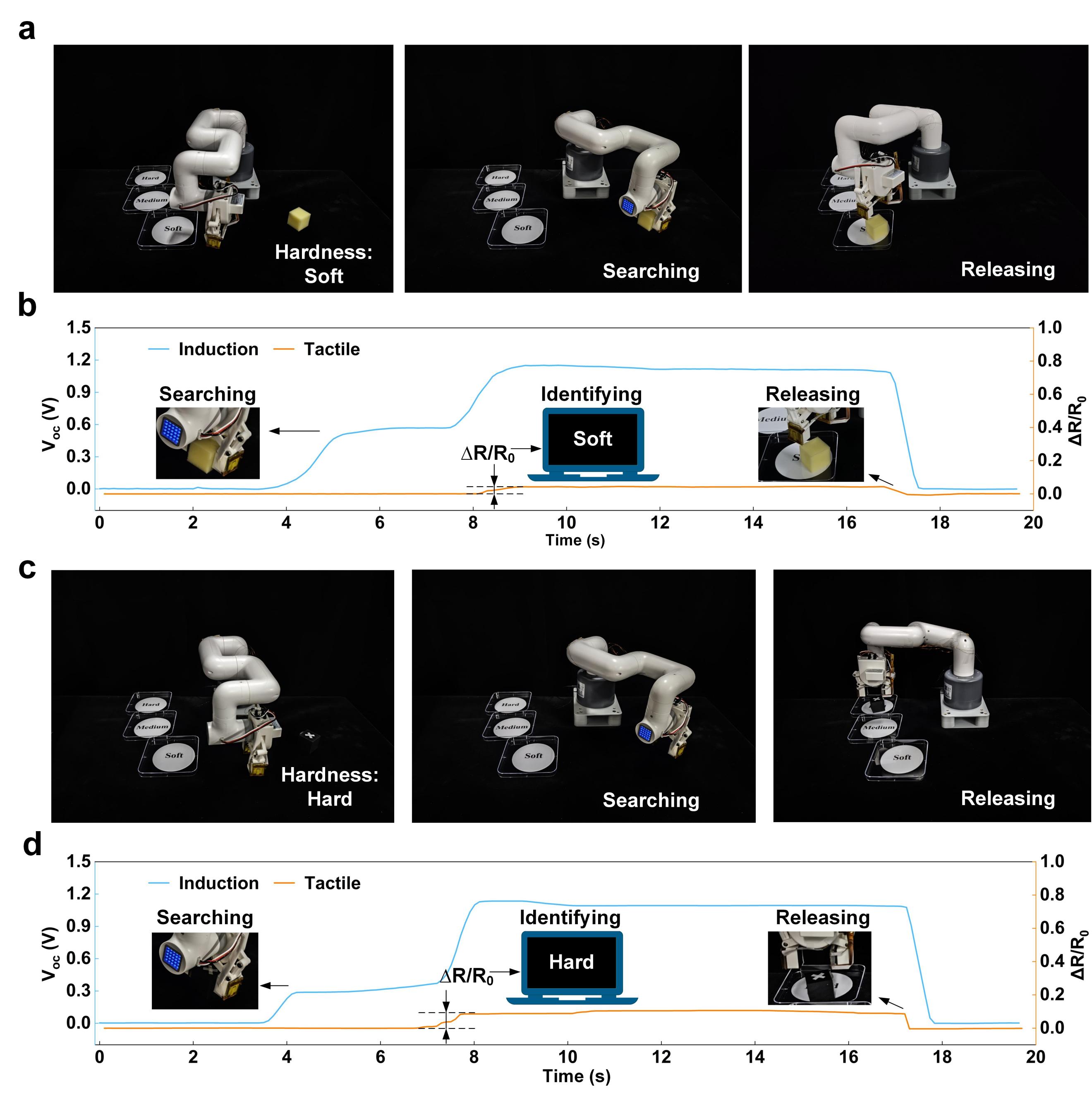


Figure S19. **a** Grasping, identification, and placement of L-Sponge at a specific position. **b** Real-time signals during the grasping, identification, and release of L-Sponge. **c** Grasping, identification, and placement of rubber at a specific position. **d** Real-time signals during the grasping, identification, and release of rubber.


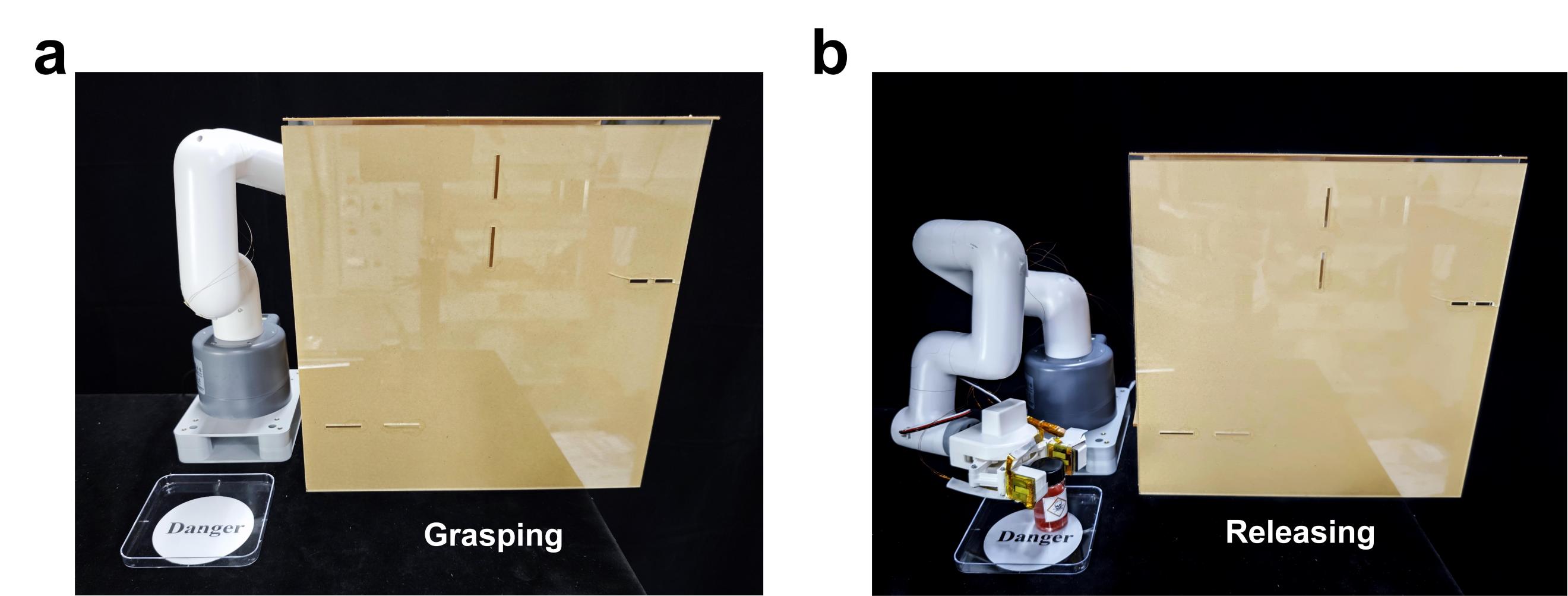


Figure S20. Vision-free grasping of hazardous objects by the IGR.


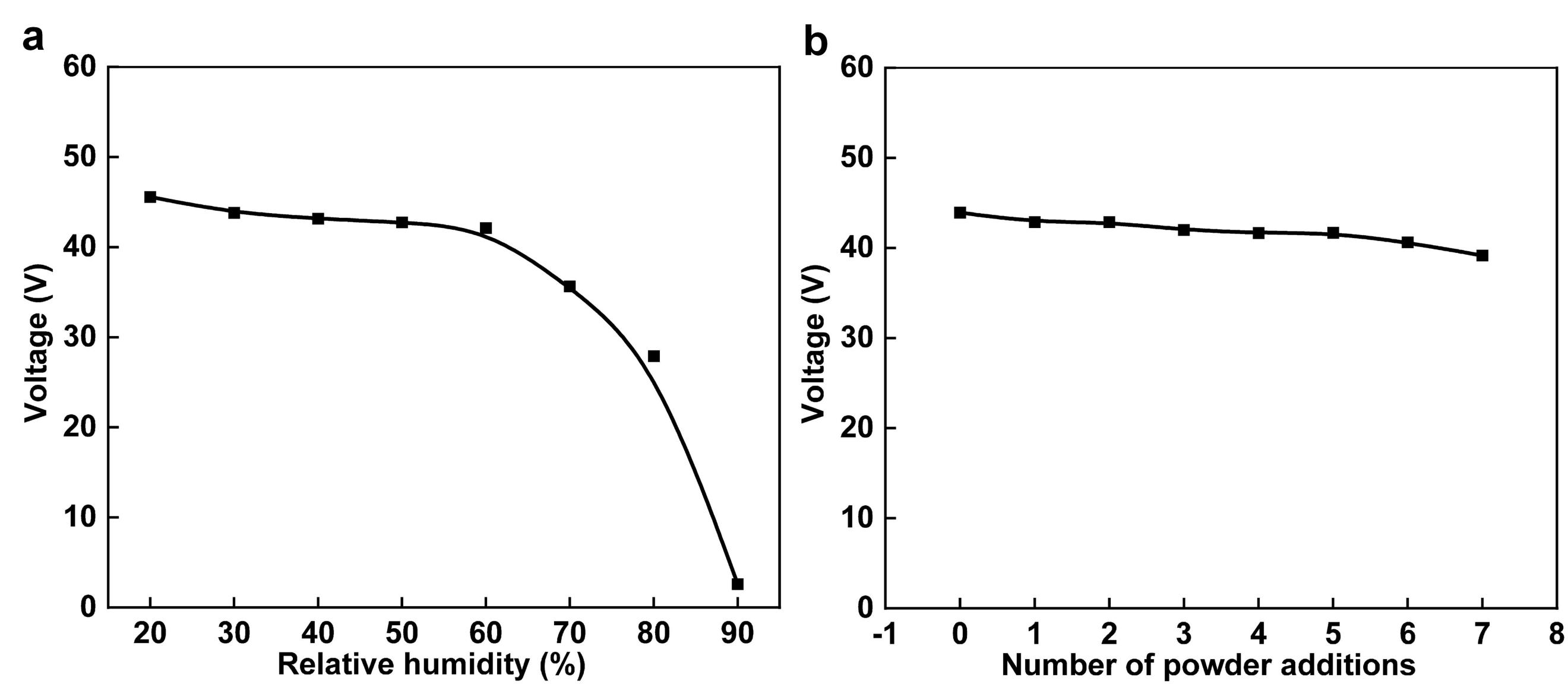


Figure S21. **a** Performance of the non‑contact induction unit under various humidity levels. **b** Performance of the non‑contact induction unit under different powder addition cycles.


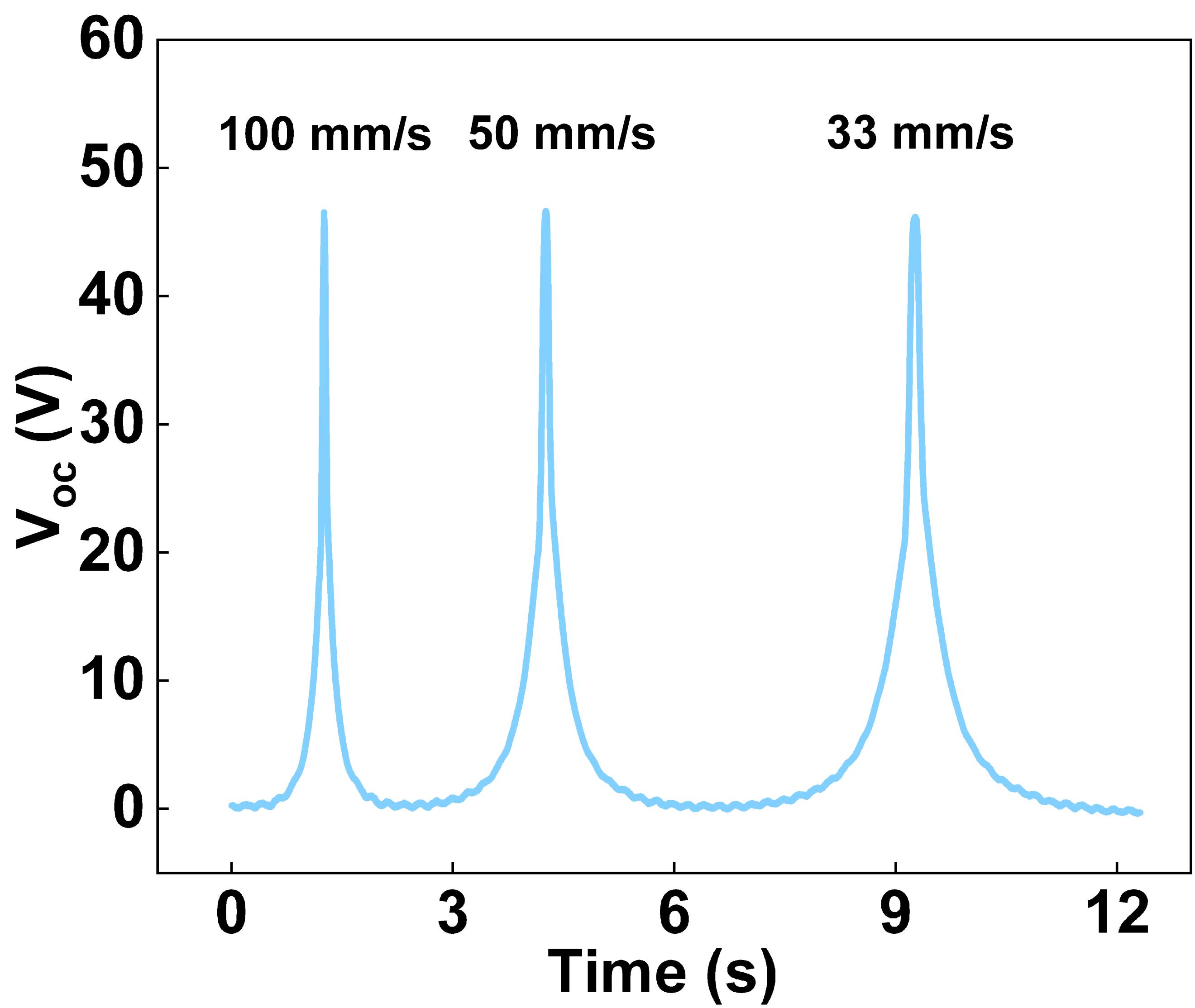


**Figure S22.** Stability of the voltage signal under different approach speeds.

When the surface charge of the sensor stabilizes, the approach speed does not affect the voltage value. The approach speed determines the rate of charge transfer, which in turn affects the current. To verify the stability of the voltage under different approach speeds, we supplemented the corresponding voltage variation curves (Figure S22). The voltage output of the non-contact induction unit is unaffected by the approach speed, as demonstrated at 100, 50, and 33 mm/s.


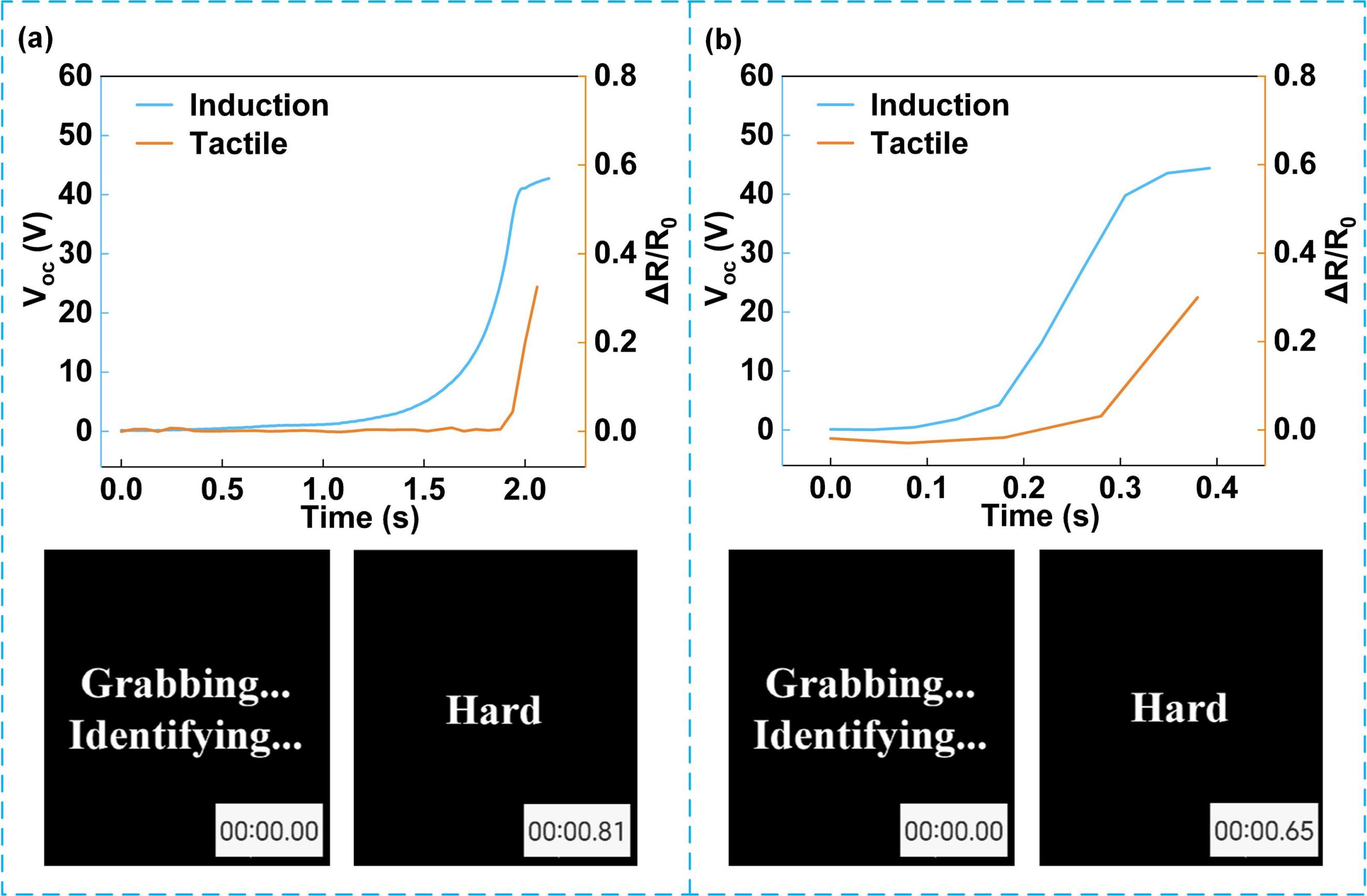


**Figure S23.** Signal demonstration and system judgment time difference under different approach speeds. **a** Signal and judgment results at an approach speed of 50 mm/s. **b** Signal and judgment results at an approach speed of 250 mm/s.

When the approach speed was 50 mm/s, the time difference between the non‑contact feedback (grasping) and the contact feedback (hardness) was 0.81 s (Figure S23a). Even when the approach speed reached the extreme limit of 250 mm/s, the non-contact and contact signals remained free from mutual interference, with the only exception being that the time difference in the recognition results did not shrink proportionally (Figure S23b). Although the delay is largely attributed to the computational processing in MATLAB, the recognition results are still correct. This latency mainly originates from the processing speed of the overall system.


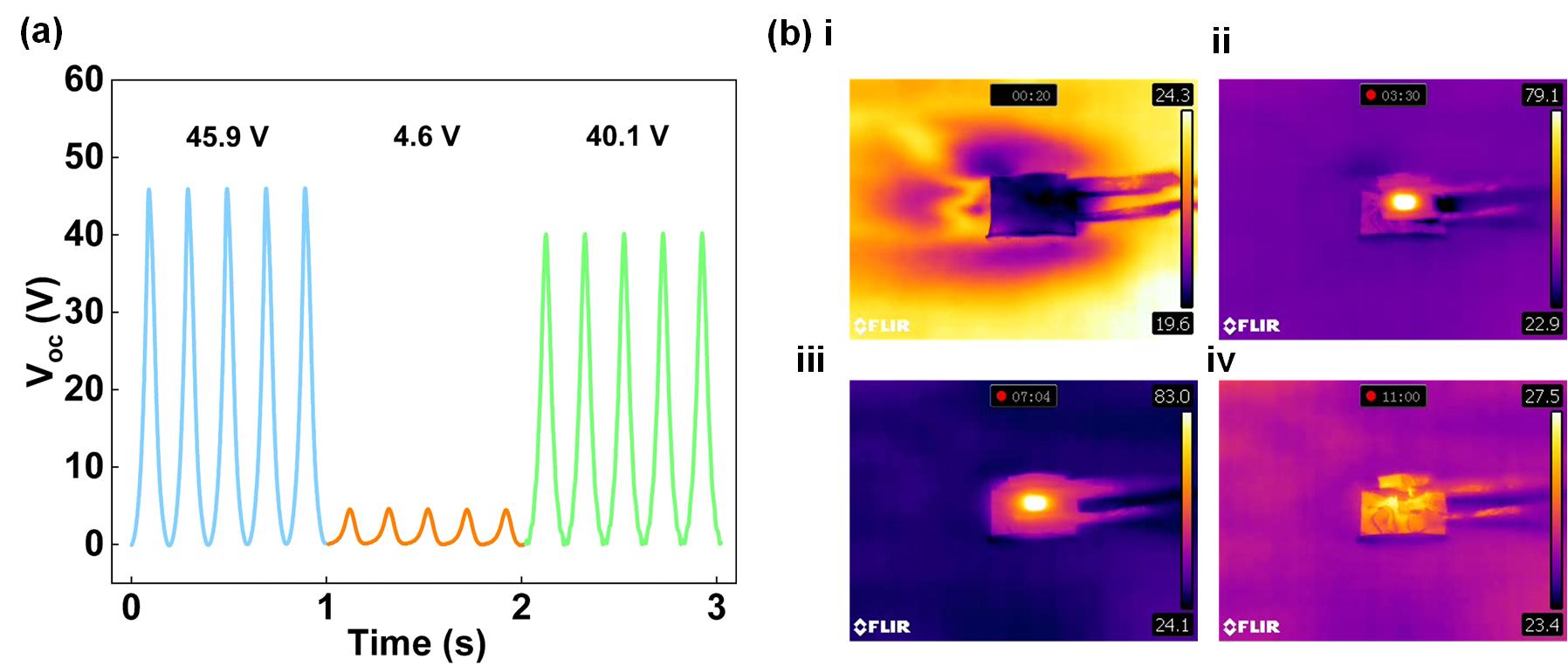


**Figure S24. a** Performance of the non-contact induction unit under three conditions: pristine state, high-humidity environment, and after dehumidification recovery. **b** Demonstration of the dehumidification effect achieved by the electrothermal property of the sensor.

The original output of the TENG was 46 V. When the TENG was placed in a high-humidity environment, its performance degraded to approximately 5 V. After evaporating the moisture using the electrothermal heating property of the sensor, a certain amount of charge re-accumulated on the triboelectric material surface, and the TENG performance recovered to 40.1 V, corresponding to a recovery rate of approximately 86% (Figure S24a).

Under high-humidity conditions, the surface temperature of the sensor was lower than the ambient temperature (Figure S24b i). Subsequently, the sensor was heated to 80℃ using electrothermal heating, a temperature that ensures the device is not damaged (Figure S24b ii) and is more suitable for normal operation. After approximately 7 minutes, the moisture on the TENG surface was evaporated (Figure S24b iii). Finally, the sensor was cooled to room temperature (Figure S24b iv).


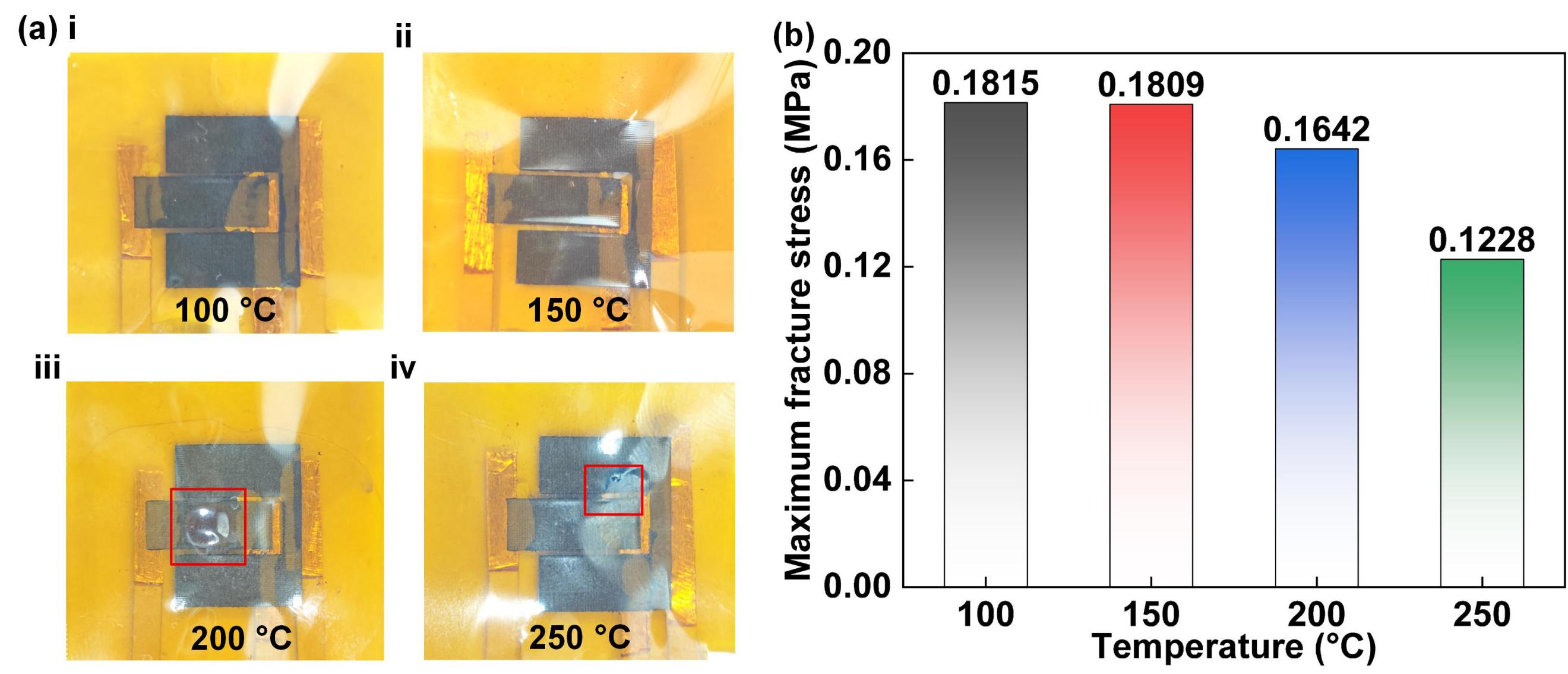


**Figure S25.** Effect of different heating temperatures on the device. **a** Degree of damage to the F-PI film observed at different temperatures. **b** Maximum fracture stress of Ecoflex after exposure to different heating temperatures.

To investigate the effect of different electrothermal heating temperatures on the device, four samples were prepared. Each sample was heated to 100℃, 150℃, 200℃, and 250℃, respectively, and maintained at the target temperature for one minute. Figure S25a shows the degree of damage to each device. The devices were unaffected by heating at 100℃ and 150℃. When the temperature reached 200℃, the PI film exhibited deformation and bubble formation. The overall structure of the device was partially damaged but remained functional. At 250℃, localized carbonization and combustion occurred in the PI film due to heat concentration, resulting in structural failure of the device.

After the heating test, the devices treated at different temperatures were mounted on a tensile testing machine. Tensile tests revealed the effect of high temperature on the structural strength of Ecoflex. When the temperature exceeded 200℃, the elevated temperature reduced the fracture strength of Ecoflex. At 250℃, the fracture strength of Ecoflex decreased further (Figure S25b). Based on the thermal response of F-PI and Ecoflex, the sensor should operate at temperatures below 200℃.

**Table S1.** Comparison of tactile perception sensitivity and detection range.

| References | Classification | Sensitivity  (kPa^-1^) | Maximum pressure range (kPa) |
| --- | --- | --- | --- |
| 1 | Functional layer | 3.42 | 30 |
| 2 | Functional layer | 0.1 | 30 |
| 3 | Functional layer | 1.43 | 71 |
| 4 | Piezoresistance | 4.1 | 41 |
| 5 | Piezoresistance | 34.15 | 224 |
| 6 | Piezoresistance | 10.49 | 140 |
| 7 | Microstructural | 4 | 7.2 |
| 8 | Microstructural | 9.21 | 100 |
| 9 | Capacitance | 14 | 200 |
| 10 | Capacitance | 7.1 | 100 |
| 11 | Capacitance | 2.69 | 50 |
| This work | Piezoresistance | 11.2 | 180 |

**Table S2.** Comparison of the performance and detection distance of the non-contact induction unit.

| References | Classification | Area-normalized performance (V/cm^2^) | Maximum detection distance  (mm) |
| --- | --- | --- | --- |
| 2 | Functional layer | 2.26 | 25 |
| 12 | Functional layer | 6.25 | 270 |
| 13 | Polymer | 0.21 | 100 |
| 14 | Polymer | 4 | 80 |
| 1 | Microstructural | 4 | 20 |
| 15 | Microstructural | 2.52 | 300 |
| 16 | Microstructural | 4.22 | 140 |
| 3 | Material modification | 3.81 | 50 |
| 17 | Material modification | 0.21 | 80 |
| 18 | Material modification | 14.31 | 30 |
| 19 | Material modification | 8 | 80 |
| This work | Material modification | 46 | 110 |

**Table S3.** Comparison of tactile perception response speed.

| References | Classification | Response speed  (ms) |
| --- | --- | --- |
| 1 | Functional layer | 120 |
| 2 | Functional layer | 10 |
| 3 | Functional layer | / |
| 4 | Piezoresistance | 0.4 |
| 5 | Piezoresistance | 70 |
| 6 | Piezoresistance | 5.6 |
| 7 | Microstructural | 80 |
| 8 | Microstructural | 34 |
| 9 | Capacitance | 70 |
| 10 | Capacitance | 500 |
| 11 | Capacitance | 100 |
| This work | Piezoresistance | 10 |

**Table S4.** Comparison of sensor complexity (layer count).

| References | Classification | number of structural layers |
| --- | --- | --- |
| 1 | Functional layer | 4 |
| 2 | Functional layer | 4 |
| 3 | Functional layer | 3 |
| 4 | Piezoresistance | 8 |
| 5 | Piezoresistance | 3 |
| 6 | Piezoresistance | 3 |
| 7 | Microstructural | 7 |
| 8 | Microstructural | 5 |
| 9 | Capacitance | 3 |
| 10 | Capacitance | 5 |
| 11 | Capacitance | 3 |
| 12 | Functional layer | 3 |
| 13 | Polymer | 3 |
| 14 | Polymer | 12 |
| 15 | Microstructural | 4 |
| 16 | Microstructural | 4 |
| 17 | Material modification | 2 |
| 18 | Material modification | 4 |
| 19 | Material modification | 3 |
| This work | Piezoresistance | 2 |

**References**

[1] W. Liu, Y. Duo, J. Liu, F. Yuan, L. Li, L. Li, G. Wang, B. Chen, S. Wang, H. Yang, Y. Liu, Y. Mo, Y. Wang, B. Fang, F. Sun, X. Ding, C. Zhang, L. Wen, *Nat. Commun.* **2022**, *13*, 5030.

[2] W. Liu, Y. Duo, X. Chen, B. Chen, T. Bu, L. Li, J. Duan, Z. Zuo, Y. Wang, B. Fang, F. Sun, K. Xu, X. Ding, C. Zhang, L. Wen, *Adv. Funct. Mater.* **2023**, *33*, 2306368.

[3] F. Dong, Q. Peng, G.-A. Yu, H. Du, W. Sha, P. Li, Y. Liu, H. Cai, T. Du, M. Xu, *Small* 2025,21, 2503393.

[4] Y. Qiu, F. Wang, Z. Zhang, K. Shi, Y. Song, J. Lu, M. Xu, M. Qian, W. Zhang, J. Wu, Z. Zhang, H. Chai, A. Liu, H. Jiang, H. Wu, *Sci. Adv.* **2024**, *10*, eadp0348.

[5] S. Han, X. Zhi, Y. Xia, W. Guo, Q. Li, D. Chen, K. Liu, X. Wang, *Small* **2023**, *19*, 2301593.

[6] Y. Li, G. Matsumura, Y. Xuan, S. Honda, K. Takei, *Adv. Funct. Mater.* **2024**, *34*, 2313824.

[7] S. He, J. Dai, D. Wan, S. Sun, X. Yang, X. Xia, Y. Zi, *Sci. Adv.* **2024**, *10*, eado6793.

[8] Y. Liu, J. Wang, T. Liu, Z. Wei, B. Luo, M. Chi, S. Zhang, C. Cai, C. Gao, T. Zhao, S. Wang, S. Nie, *Nat. Commun.* **2025**, *16*, 383.

[9] H. Niu, H. Li, Q. Zhang, E.-S. Kim, N.-Y. Kim, Y. Li, *Small* **2024**, *20*, 2308127.

[10] S. Li, S. Chen, L. Yang, Y. Guo, K. Tan, S. Liu, J. Qiu, B. Yuan, J. Yu, *Nano Energy* **2023**, *118*, 108946.

[11] Y. Lv, Z. Ma, J. Duan, G. Sun, P. Wang, S. Qu, F. Liu, C. Meng, X. Lin, T. Liu, S. Guo, *InfoMat* **2025**, *7*, e70041.

[12] J. Ma, J. Zhu, P. Ma, Y. Jie, Z. L. Wang, X. Cao, *ACS Energy Lett.* **2020**, *5*, 3005.

[13] S. Peng, Y. Feng, Y. Liu, M. Feng, Z. Wu, J. Cheng, Z. Zhang, Y. Liu, R. Shen, D. Wang, *Nano Energy* **2022**, *104*, 107899.

[14] G. Ye, Q. Wu, Y. Chen, X. Wang, Z. Xiang, J. Duan, Y. Wan, P. Yang, *Adv. Fiber Mater.* **2024**, *6*, 1874.

[15] S. Kang, S. Noh, J. Shin, S. Jhee, N.-I. Kim, J.-H. Ryou, J. S. Kim, *Chem. Eng. J.* **2026**, *527*, 171964.

[16] K. Shrestha, S. Sharma, G. B. Pradhan, T. Bhatta, P. Maharjan, S. S. Rana, S. Lee, S. Seonu, Y. Shin, J. Y. Park, *Adv. Funct. Mater.* **2022**, *32*, 2113005.

[17] F. Yuan, S. Liu, J. Zhou, S. Wang, Y. Wang, S. Xuan, X. Gong, *Nano Energy* **2021**, *86*, 106071.

[18] Q. Zhang, J. Ma, Z. Yin, Y. Feng, D. Song, F. Gao, X. Cao, T. Jiao, *Chem. Eng. J.* **2025**, *525*, 170084.

[19] X. Li, P. Wang, C. Zong, H. Niu, P. Xu, Y. Yu, Y. Li, G. Shen, *Nano Energy* **2025**, *140*, 110995.
